# Persisting Viral Sequences Shape Microbial CRISPR-based Immunity

**DOI:** 10.1371/journal.pcbi.1002475

**Published:** 2012-04-19

**Authors:** Ariel D. Weinberger, Christine L. Sun, Mateusz M. Pluciński, Vincent J. Denef, Brian C. Thomas, Philippe Horvath, Rodolphe Barrangou, Michael S. Gilmore, Wayne M. Getz, Jillian F. Banfield

**Affiliations:** 1 Biophysics Graduate Group, University of California, Berkeley, California, United States of America; 2 Departments of Ophthalmology and Microbiology and Immunobiology, Harvard Medical School, Boston, Massachusetts, United States of America; 3 Department of Plant and Microbial Biology, University of California, Berkeley, California, United States of America; 4 Department of Environmental Science, Policy and Management, University of California, Berkeley, California, United States of America; 5 Division of Epidemiology, School of Public Health, University of California, Berkeley, California, United States of America; 6 DuPont Nutrition and Health, Dange-Saint-Romain, France; 7 DuPont Nutrition and Health, Madison, Wisconsin, United States of America; 8 Microbial Sciences Initiative, Harvard University, Cambridge, Massachusetts, United States of America; 9 Department of Earth and Planetary Sciences, University of California, Berkeley, California, United States of America; University of Zurich and Swiss Institute of Bioinformatics, Switzerland

## Abstract

Well-studied innate immune systems exist throughout bacteria and archaea, but a more recently discovered genomic locus may offer prokaryotes surprising immunological adaptability. Mediated by a cassette-like genomic locus termed Clustered Regularly Interspaced Short Palindromic Repeats (CRISPR), the microbial adaptive immune system differs from its eukaryotic immune analogues by incorporating new immunities unidirectionally. CRISPR thus stores genomically recoverable timelines of virus-host coevolution in natural organisms refractory to laboratory cultivation. Here we combined a population genetic mathematical model of CRISPR-virus coevolution with six years of metagenomic sequencing to link the recoverable genomic dynamics of CRISPR loci to the unknown population dynamics of virus and host in natural communities. Metagenomic reconstructions in an acid-mine drainage system document CRISPR loci conserving ancestral immune elements to the base-pair across thousands of microbial generations. This ‘trailer-end conservation’ occurs despite rapid viral mutation and despite rapid prokaryotic genomic deletion. The trailer-ends of many reconstructed CRISPR loci are also largely identical across a population. ‘Trailer-end clonality’ occurs despite predictions of host immunological diversity due to negative frequency dependent selection (kill the winner dynamics). Statistical clustering and model simulations explain this lack of diversity by capturing rapid selective sweeps by highly immune CRISPR lineages. Potentially explaining ‘trailer-end conservation,’ we record the first example of a viral bloom overwhelming a CRISPR system. The polyclonal viruses bloom even though they share sequences previously targeted by host CRISPR loci. Simulations show how increasing random genomic deletions in CRISPR loci purges immunological controls on long-lived viral sequences, allowing polyclonal viruses to bloom and depressing host fitness. Our results thus link documented patterns of genomic conservation in CRISPR loci to an evolutionary advantage against persistent viruses. By maintaining old immunities, selection may be tuning CRISPR-mediated immunity against viruses reemerging from lysogeny or migration.

## Author Summary

Most microbes appear unculturable in the laboratory, limiting our knowledge of how virus and prokaryotic host evolve in natural systems. However, a genomic locus found in many prokaryotes, CRISPR, may offer cultivation-independent probes of virus-microbe coevolution. Utilizing nearby genes, CRISPR can serially incorporate short viral and plasmid sequences. These sequences bind and cleave cognate regions in subsequent viral and plasmid insertions, conferring adaptive anti-viral and anti-plasmid immunity. By incorporating sequences undirectionally, CRISPR also provides timelines of virus-prokaryote coevolution. Yet, CRISPR only incorporates 30–80 base-pair viral sequences, leaving incomplete coevolutionary recordings. To reconstruct the missing coevolutionary dynamics shaping natural CRISPRs, we combined metagenomic reconstructions with population-scale mathematical modeling. Capturing rare and rapid sweeps of CRISPR diversity by highly immune lines, mathematical modeling explains why naturally reconstructed CRISPR loci are often largely identical across a population. Both model and experiment further document surprising proliferations of old viral sequences against which hosts had preexisting CRISPR immunity. Due to these deadly blooms of ancestral viral elements, CRISPR's conservation of old immune sequences appears to confer a selective advantage. This may explain the striking immunological memory documented in CRISPR loci, which occurs despite rapid viral mutation and despite rapid deletions in prokaryotic genomes.

## Introduction

Innate immune systems with built-in self/non-self recognition mechanisms have long been known to protect prokaryotic genomes against insertions of foreign DNA [1]. For example, well-studied restriction-modification systems often preserve genomic integrity by methylating prokaryotic DNA, enabling prokaryotes to recognize and cleave unmethylated foreign DNA [2]. Yet, the foreign DNA attacking prokaryotes includes the most abundant and rapidly diversifying members of the biosphere, viruses [3]. With viruses quickly evolving counter-strategies against prokaryotic immune systems, prokaryotes require immunological plasticity to keep pace. Here we computationally predict and directly document the evolution of an adaptive immune system that enables prokaryotes to serially acquire new immunities against diversifying viruses and plasmids. Importantly, the prokaryotic adaptive immune system is genomically encoded (i.e., hereditable) and acquires new immune elements unidirectionally, making this adaptive immune system distinct from its eukaryotic analogues [4], [5].

The microbial adaptive immune system is mediated by a genomic locus termed Clustered Regularly Interspaced Short Palindromic Repeats (CRISPR). CRISPR loci have been found in approximately 45% of sequenced bacteria and over 90% of sequenced archaea [6], [7]. Utilizing adjacently encoded CRISPR-associated (Cas) proteins [8], CRISPR loci incorporate short 21–72 base-pair sequences from targeted regions in invading viruses and plasmids [4], [6], [7], [9], [10], [11], [12]. Once transcribed and processed into CRISPR RNAs, these viral and plasmid-derived sequences confer sequence-specific immunity by binding and cleaving cognate viral and plasmid regions during subsequent genomic invasions [13], [14].

The viral and plasmid binding sequences incorporated into host CRISPR loci are termed ‘spacers,’ reflecting their addition interspacing highly synonymous 23–47 base-pair sequences, termed ‘repeats’ [4], [15], [16]. Correspondingly, the targeted viral and plasmid sequences are known as ‘proto-spacers’ [4], [15]. With spacer immunity specific to a matching proto-spacer sequence, viruses can escape CRISPR targeting by mutating their proto-spacers or by mutating nearby proto-spacer adjacent motifs (PAMs), regions which likely act as recognition sites for the CRISPR/Cas machinery [4], [15]. Natural selection favors the emergence of viruses with mutations in CRISPR-targeted regions, leading to a coevolutionary arms race [17] as hosts incorporate new spacers to combat viral adaptations [8], [15], [18]. Coevolutionary arms races have been well-documented in other virus-microbe systems [19], [20], [21], [22], [23]. Yet, unlike previously studied coevolutionary wars, CRISPR recorded arms races naturally differentiate current host adaptations from previous host adaptations. This is because new spacers are added unidirectionally, adjacent to a leader sequence at a single end of the locus termed the ‘leader-end.’ Previously acquired spacers are also commonly maintained, leaving a cassette-like recording of current (i.e., spacers closest to the leader-end) and past (i.e., spacers farther from the leader-end) adaptations. Partial timelines of coevolution can thus be constructed for host and viral species refractory to laboratory challenge experiments [3].

Previously, we described one CRISPR recording through metagenomic reconstructions of the CRISPR loci sampled from floating microbial biofilms in an acid mine drainage (AMD) system [24]. The prime advantage of probing these generally closed, acidophilic environments is that they are dominated by relatively few species [25]. Our AMD research targeted the extremophilic archaeon I-plasma [18]. Growing in an AMD biofilm matrix at temperatures ranging from approximately 30° to 48° Celsius and pHs ranging from approximately 0.3 to 1.2, I-plasma is one of around 12 species in the acidophilic order Thermoplasmatales [26], [27]. Reconstructing the CRISPR loci of I-plasma, we noted that the newest, leader-end spacers emerged highly diverse and cell-specific. In contrast, the trailer-end spacers (i.e., the oldest spacers found farthest from the leader sequence) were highly clonal population-wide, matching earlier observations of trailer-end clonality in acidophilic *Leptospirillum* bacteria [28] and more recent observations in bacterial *Escherichia coli* and archaeal *Sulfolobus islandicus* [29], [30].

Surprisingly, I-plasma's trailer-end spacers appeared conserved despite appearing to provide no immunity against current viruses (Figure S1). In reconstructions (∼20-fold coverage) of the I-plasma locus in the AMD biofilm, only newly acquired leader-end CRISPR spacers matched currently sampled viruses, implying that previously targeted viral sequences had since evolved or disappeared. Similarly, laboratory challenge experiments [4], [15] document rapid viral evolution in the face of CRISPR targeting.

Here we sought to understand why trailer-end spacers are often conserved despite failing to confer immunity against current viruses. Trailer-end conservation is especially surprising in light of the genomic compactness of Bacteria and Archaea, whose genomes rarely exceed 13MB [31]. Prokaryotes have also been shown to delete genetic material approximately ten times as frequently as they insert [32]. With a bias toward genomic deletions, we hypothesized that bacteria and archaea would only preserve CRISPR's genetic material if natural selection favored it.

To find and probe the selection pressure driving the preservation of CRISPR trailer-ends, we combined metagenomic reconstructions of CRISPR loci across a multi-year period with a population-genetic mathematical model of virus-CRISPR dynamics in a natural system. Three previous studies have constructed mathematical models of virus-host dynamics in the CRISPR system [33], [34], [35], but none were built to explain why CRISPR loci emerge with both trailer-end clonality and trailer-end conservation. Building a model in which CRISPR locus length is an emergent property of the model parameters, we probe whether tuning parameters to increase trailer-end conservation increases prokaryotic fitness even when viruses mutate rapidly. We further capture the dynamics through which the trailer-ends of CRISPR loci are purged of spacer diversity.

### Model

A population-genetic model (see Text S1 for the full algorithm) was built to analyze how the intracellular processes of CRISPR and virus mutation drive the long-term development of natural CRISPR loci captured via metagenomic analysis. For simplicity, the model restricts its study of host and viral genomes to monitoring host spacers and viral proto-spacers. All other elements in the genomes are ignored. Host and viral populations are then divided into ‘strains’: all hosts sharing the same ordered set of spacers are assigned to a single host strain while all viruses with identical proto-spacers are assigned to a single viral strain (Figure S2). Each strain's cumulative frequency is tracked across thousands of iterations, as mutations alter host immunity and viral infectivity.

The iterations of the model are not directly dependent on time. Each iteration is instead defined to be the period of variable duration in which a large, preset number of virus-host interactions occurs (Table 1). During each virus-host interaction, one of two possible outcomes generally occurs. If the host and viral strains share a spacer, the host survives and the virus is cleared. Conversely, if no spacer is shared, the virus kills the host and the virus survives. Of course, exceptions to both of these situations are allowed in the model. Hosts are given a small probability of surviving even when lacking spacers against an invading virus (Table 1). Further, CRISPR is given a small probability of failing to provide immunity even when a host spacer matches an infecting virus' proto-spacer (Table 1). This failure rate has been measured in viral plaquing assays conducted by two independent groups [4], [36].

**Table 1:** Table of parameters used in model.

Symbol	Value (Range Probed)	Description
*K*	10^6^ (10^5^–10^8^)	Interactions per iteration.
*S*	50 (1–300)	Fixed number of proto-spacers per viral genome.
*P*_v_mut_	.003 (10^−4^–3•10^−3^)	Probability that viruses mutate a random proto-spacer in an interaction. For bacteria and DNA-based viruses this has been measured at ∼.003 mutations per genome per replication [72].
*P*_b_add_	8•10^−6^ (10^−6^–10^−4^)	Probability that hosts unidirectionally add a random spacer in an interaction, as measured in CRISPR laboratory experiments [10]. With 10^6^ interactions per iteration, numerous (*e.g.*, 8) strains add new spacers per iteration, causing clonal interference (‘kill the winner’) and multiple-mutation driven sweeps.
*P*_b_lose_	0 (0–1)	Expected frequency of spacer additions in which hosts delete a random spacer block.
*f(n)*	10^(−4+n)^ n>0 1–10^−9^ n = 0	Given *n* shared spacers, the probability a virus-host interaction is productive (*i.e.*, virus lives and host dies). When n = 0, f is set to an extremely small but still positive number to prevent host extinction.
*i_B_*	0.1 (.01–0.5)	Fraction of parent strain's frequency that each host mutant is initialized with. Because CRISPR immunity is genetic, fitness is inherited from parent strains.
*i_V_*	0.1 (.01–0.5)	Fraction of parent strain's frequency that each viral mutant is initialized with.
*G*	3 (0–3)	Average of Poisson-distributed clearance-free emergence iterations given to each new host and viral mutant strain.
*V*_min_freq_	10^−6^ (10^−8^–10^−3^)	Frequency threshold below which viral strains beyond their emergence iterations are cleared.
*B*_min_freq_	10^−6^ (10^−8^–10^−3^)	Frequency threshold below which host strains beyond their emergence iterations are cleared.
*V*_list_max_	300 (100–5000)	Maximum number of surviving viral strains beyond their emergence iterations.
*B*_list_max_	300 (100–5000)	Maximum number of surviving host strains beyond their emergence iterations.

With a large number of interactions per iteration, virus-host interactions are assumed to be well-mixed and distributed according to strain frequencies. Since viruses are most likely to encounter high-frequency host strains, this selects for the viral lines that can kill the dominant hosts, resulting in negative frequency-dependent selection, a process termed ‘kill the winner’ in microbial ecology [37]. During some interactions, stochastic mutations create new host and viral strains, as hosts unidirectionally add spacers and viruses mutate random proto-spacers. Old host and viral strains are simultaneously depressed in frequency and driven extinct when no longer immune and infective, respectively. At the end of an iteration, the model takes a metagenomic snapshot of the surviving host and viral populations. We analyzed these snapshots across model iterations to capture patterns of CRISPR-driven immunity as they emerge.

#### Model assumptions

Here we describe the main assumptions of the model; a more in-depth analysis of each model assumption can be found in the Supplementary Information (Text S2). First, the model assumes that virus and host populations do not go irreversibly extinct. With host and viral populations continually extant, in each iteration the model can simply wait until any preset number of virus-host interactions occurs. We can thus define iterations to be the variable duration period in which such a preset number of interactions occurs. Empirical support for assuming the long-run coexistence of virus and host in natural environments comes from two metagenomic studies. In the first study, Rodriguez-Brito *et al.*, [38] recovered consistently high amounts of virus and host genomes in four aquatic regions across a year-long period. Similarly, in the experimental part of our study, we reconstructed the relative abundances of CRISPR loci and viruses in an acid mine drainage system across the last two years of our six-year metagenomic time series experiment. In each sampling, both host and viral genomes were recovered.

Large microbial population sizes limit the effect of sampling noise in modulating the frequencies (genetic drift) of established strains in our model. But since new mutants arise at low frequencies, we incorporated demographic stochasticity in their ability to establish (*i.e.*, avoid extinction due to a low initial frequency). We did so by allowing new mutants randomly distributed ‘emergence periods’ during which they were not subject to the model's clearance of low-frequency strains. All strains, excluding new mutants in their randomly-sized emergence periods, are cleared when their frequencies drop below a threshold, effecting mutation-selection balance and preventing the model from accumulating an uncontrollable number of strains as new mutants are created. Thus, without the randomness component, the emergence period allows new mutants a chance to reach ‘establishment frequencies,’ after which each mutant can compete in the model solely via its CRISPR-determined fitness.

By increasing the rate at which viable mutants establish, the emergence period increases competition between distinct spacer-adding lines (clonal interference). This promotes ‘kill the winner’ dynamics, making it harder for individual lines to sweep. Despite this increase in competition among beneficial mutants, below we capture losses of trailer-end diversity and rapid selective sweeps. To assure that these results also occur without the emergence period, we tested the model without an emergence period and found both trailer-end clonality and stochastic sweeps (Figure S3).

## Results

### CRISPR trailer-end conservation across multi-year reconstructions

Before analyzing the selective pressure responsible for trailer-end conservation in the single snapshot of CRISPR loci shown in Figure S1, we first sought to rigorously determine whether hosts actually preserve CRISPR trailer-ends across evolutionary timescales. To do so, we metagenomically tracked CRISPR spacer content and structure in a natural system over a six-year period. Our analyses focused on an archaeal G-plasma population and abundant viruses that target it. Like I-plasma, G-plasma is a species in the order Thermoplasmatales [26], [27]. Yet, G-plasma and I-plasma are sufficiently divergent at the rRNA gene sequence and amino acid level to be considered distinct genera [39]. Moreover, the lineages show limited genome synteny [39].

**Figure 1:**
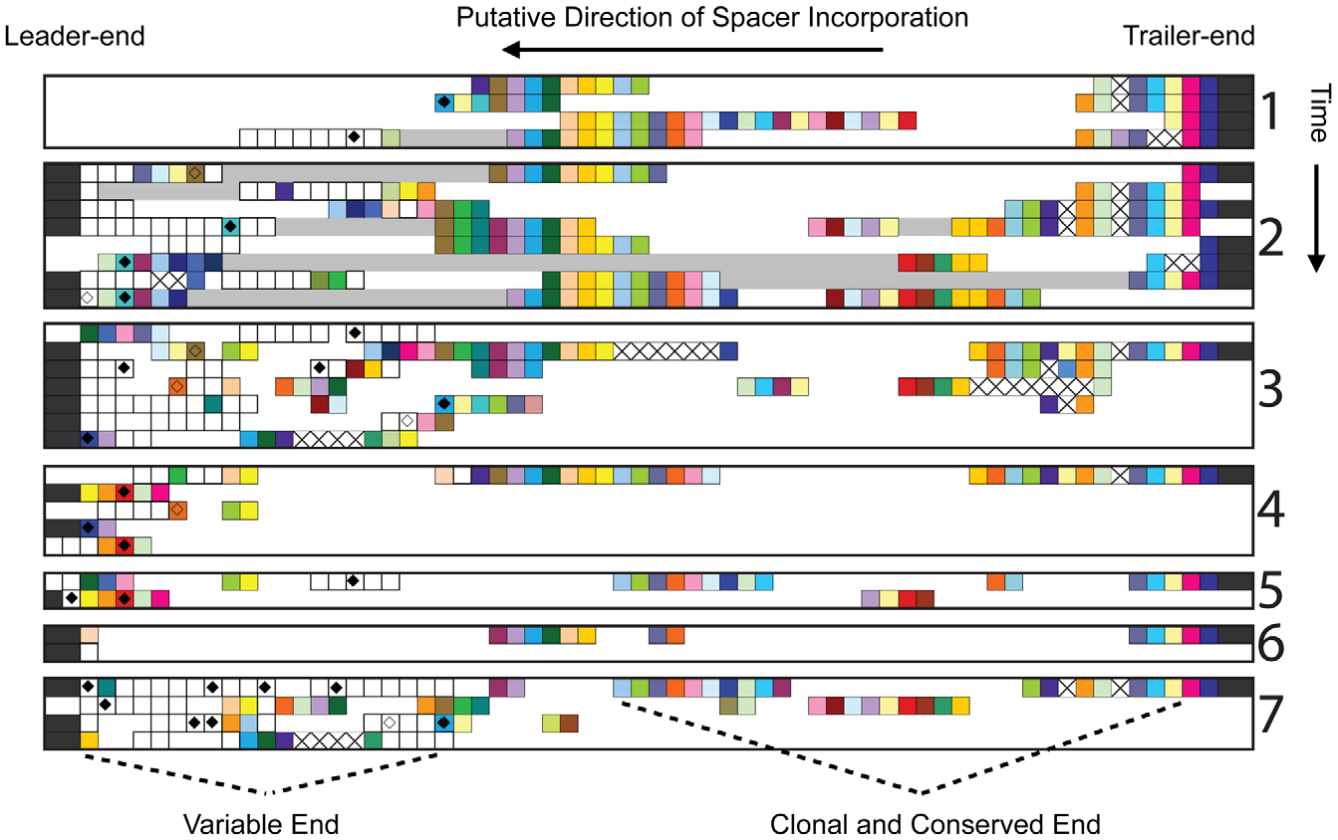
Trailer-end conservation and clonality documented in G-plasma CRISPR loci #1. Metagenomic reconstructions of the first CRISPR locus of a G-plasma population sampled in 2002 (1), 2005 (2), June 2006 (3), August 2006 (4) November 2006 (5), May 2007 (6) and August 2007 (7). In each sampling, the CRISPR spacers (boxes) are aligned horizontally according to their ordering in the metagenomic reads, with CRISPR repeats removed for compactness. Overlapping 454 spacer patterns are also condensed (Methods). The left-ends are the leader-ends, where new spacers are unidirectionally incorporated. Boxes filled with the same color represent identical spacers, with two exceptions. Black-filled boxes show flanking genetic material and white-filled boxes denote cell-specific spacers found only once in the dataset. White gaps reflect unsequenced regions in the metagenomic reconstructions. When separated spacers can be linked via paired reads, the intervening region is shown as a grey bar. Boxes containing a black ‘X’ indicate probable spacer deletions. When spacers match reconstructed AMDV3b viral sequences, diamonds are inserted, with filled diamonds showing perfect matches and open diamonds reflecting imperfect matches. Trailer-end conservation (presumed immunological memory) and clonality are pronounced in this locus, with large numbers of matching spacers preserved across the six-year period. Another example of trailer-end conservation and clonality—in the CRISPR loci of archaeal I-plasma—is shown in Figure S1.

**Figure 2:**
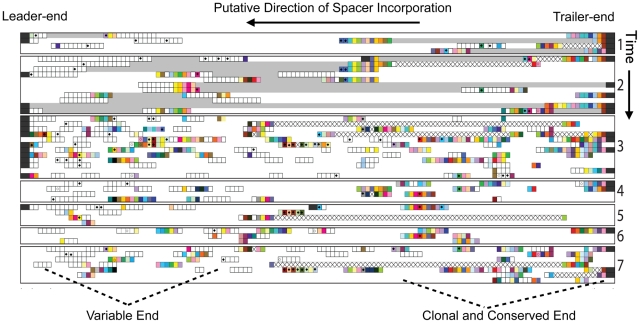
Trailer-end spacers of G-plasma CRISPR locus #2 match AMDV3b viral regions across the six-year period. Metagenomic reconstructions of the second CRISPR locus of G-plasma at the seven sampled time points. Notably, several trailer-end G-plasma spacers match reconstructed AMDV3b across all time points (filled diamonds).

To evaluate the extent to which G-plasma CRISPR locus spacers are conserved across time, we metagenomically reconstructed G-plasma CRISPR fragments seven times during the six-year study. In each sampling, the spacers in the CRISPR loci were aligned based on flanking genome sequences and paired read information (Methods). Notably, trailer-end spacers were conserved in both loci across the multi-year period (Figures 1 and 2).

Spacer preservation occurs despite deletions of single and multiple spacer-repeat units. Deletions of old spacers have also been observed in previous studies [7], [15], [16], [28]. With new spacers more likely to provide immunity against current co-evolving viruses [18], we wondered why trailer-end CRISPR spacers are maintained. To probe whether natural selection conserves old spacers to maintain immunity against persisting viruses, we used the community genomic data across time to reconstruct putative viruses throughout the multi-year period (Methods). We previously noted that the first reconstructed virus, AMDV3, targets G-plasma. We inferred G-plasma targeting by detecting matches between G-plasma's CRISPR spacers and corresponding ‘proto-spacer’ sequences in AMDV3 [18]. In the current study, a variant of AMDV3, denoted AMDV3b, was reconstructed and shown to also target G-Plasma. Importantly, each viral population is genomically heterogeneous due to single nucleotide polymorphisms (SNPs) and sequence insertions and deletions.

To test whether conserved trailer-end spacers may provide immunity to persisting viruses, we mapped G-plasma CRISPR spacers onto the reconstructed viral genomes (Methods). While most spacers shared between host and viral genomes were found at the new ends of G-plasma loci, several spacers with perfect identity to AMDV3b persist in older regions across all sampled times. The spacers matching AMDV3b are shown with black diamonds in Figure 2.

### Modeling reconstructs rapid depletions of CRISPR diversity

In addition to maintaining trailer-end spacers (‘trailer-end conservation’), reconstructed CRISPR loci show far less spacer diversity at trailer-end positions than leader-end positions (‘trailer-end clonality’). Unlike conservation, trailer-end clonality could have been expected from single time-point reconstructions, as have been reported previously [28], [29]. Yet, previous analyses could not explain the dynamics through which trailer-end clonality emerges in natural CRISPR loci. In the I-plasma locus (Figure S1), all but the four newest spacer positions are clonal population-wide, indicating a recent selective sweep by an immune host lineage. Such a selective sweep is surprising in light of the cell-specific spacer diversity at the new ends of CRISPR loci. With a spacer addition rate high enough to enable numerous lines to acquire distinct beneficial spacers before any one line has swept (*i.e.*, new-end diversity), one expects that competition between spacer-adding lines would prevent selective sweeps in a process known as clonal interference [40]. Further complicating the question of how trailer-end diversity is purged from CRISPR loci is the fact that the loss of trailer-end diversity does not have to occur via selection: it could result from the unidirectional nature of spacer addition. With spacers only incorporated at new-ends, trailer-end spacer diversity cannot increase once trailer-end positions have been filled, because no distinct spacers are incorporated there. Thus, as time progresses, all but one trailer-end lineage, the ‘coalescent,’ will necessarily go extinct even without selection, resulting in trailer-end clonality.

To ascertain whether selection drives losses of diversity at CRISPR trailer-ends despite high spacer addition rates (an average of eight spacer additions occur per iteration; see Table 1), we followed the spacer diversity of computationally reconstructed locus positions for thousands of iterations. We aimed to discover how rapidly locus positions evolved from highly polyclonal to clonal, using rapidity as a marker for sweeps. For simplicity, spacer deletions were removed from the model for this step, as we focused on the role of beneficial mutations (spacer additions) in driving losses of diversity.

**Figure 3:**
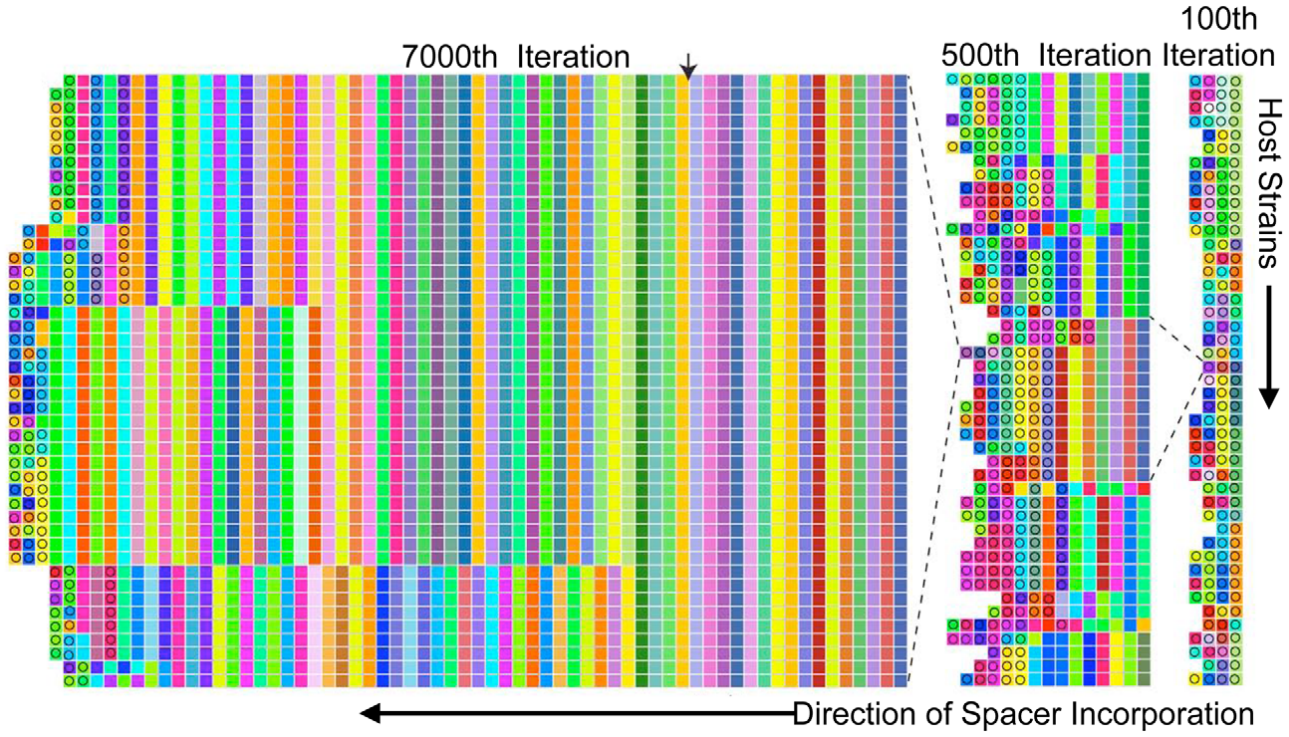
Model captures the emergence of trailer-end clonality in CRISPR loci. Computational reconstructions show the loss of trailer-end diversity from CRISPR loci. Reconstructions show the 45 most frequent host strains at the 100^th^, 500^th^ and 7000^th^ iterations of a representative simulation without spacer deletion. In each panel, the rows show distinct host strains, with their spacers allayed across the columns from right to left as in Figures 1, 2 and S1. Circles indicate spacers perfectly matching any of the 300 most frequent viral strains in that iteration. To preserve space, 128 clonal columns are removed in iteration 7000 prior to the divergence of sub-populations from a common ancestor (arrow). Notably one ancestral population still at low frequency (∼0.007 as shown in Figure S4) in the 100^th^ iteration is the common ancestor of all surviving strains.

As could be expected from the unidirectionality of spacer addition, after thousands of iterations, long-run model trajectories converge to the familiar pattern in which trailer-end spacers are clonal population-wide, while only polyclonal new-end spacers match co-evolving viruses (Figure 3 Left Panel). As in Figure S1, the majority of the locus is clonal (as noted on the figure, 128 clonal columns were removed for space conservation). Despite the eventual emergence of trailer-end clonality, CRISPR trailer-ends were initially highly diverse leader-ends (Figure 3, Right Panel and Figure S4). Interestingly, we reconstructed an intermediate stage in which the trailer-ends can be grouped into several sub-populations distinguished by their oldest spacers, indicating that gradual losses of diversity occur in the model (Figure 3 Middle Panel). Trailer-end sub-populations were similarly reported in metagenomic reconstructions from natural environments [28], [29]. By tracking the frequencies of the top 14 spacers in one of the oldest CRISPR locus positions across thousands of iterations, we further verified that spacer fixations can require thousands of iterations (Figure S5).

**Figure 4 pcbi-1002475-g004:**
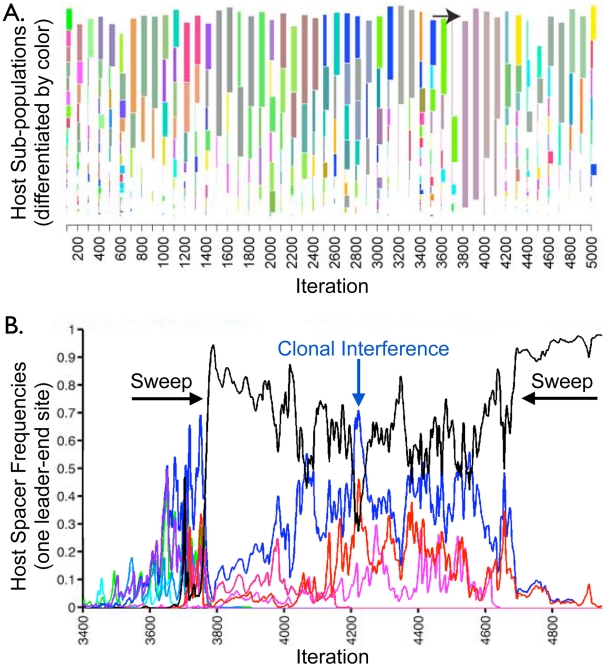
A selective sweep of spacer diversity. (A) Hosts CRISPR loci from the simulation in Figure 3 are clustered (Text S3) into distinct sub-populations every 100 model iterations to capture how trailer-end clonality emerges. Cluster heights represent the cumulative frequencies of all strains in a given cluster, cluster widths show the number of distinct strains in that cluster, and the combined height of all clusters in an iteration reflects the fraction of virus-host interactions that is immune (i.e., host mean fitness). A marked loss of host diversity occurs prior to iteration 3800 (→), after which the sweeping sub-population diversifies through distinct leader-end spacer incorporations (Figure S6). (B) The frequencies of all host spacers at a single leader-end column are tracked during the clustering-predicted sweep. A single spacer (shown in black) rapidly rises in frequency before iteration 3800 as predicted by the clustering. Subsequent ‘kill the winner’ oscillations occur before all competing hosts go extinct. A second sweep purges the remaining diversity at this locus position.

**Figure 5:**
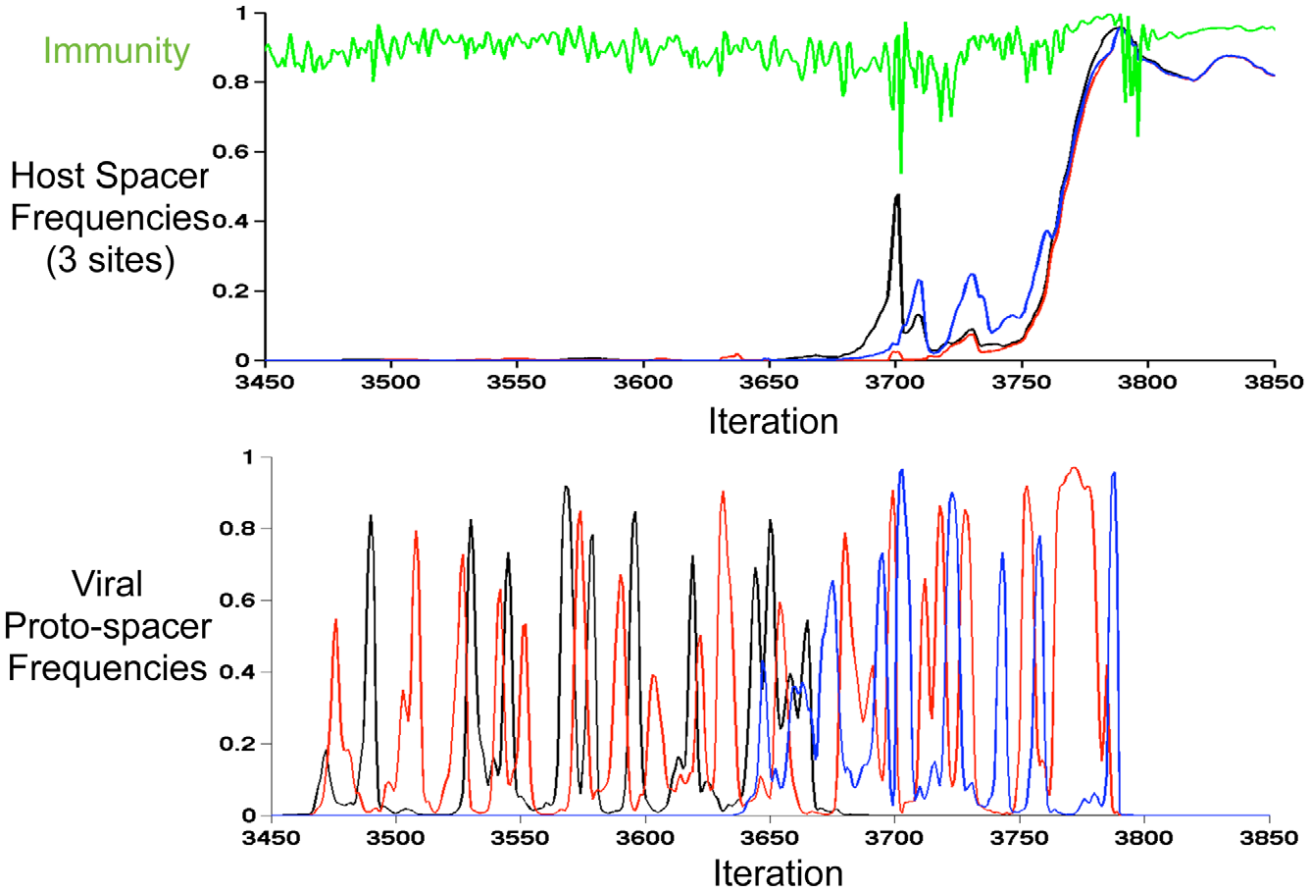
Sweep driven by spacer-mediated immunity against multiple viral sub-populations. In the upper panel, the frequency of the sweeping spacer identified in Figure 4B is again shown in black. Also tracked, are the two adjacent spacers added by the black spacer's successful host line. The frequencies of these adjacent spacers in their respective locus columns are shown in red and blue. In green, we track the fraction of immune virus-host interactions. The lower panel shows the frequencies of the three corresponding proto-spacers in the viral population. The inverse fluctuations in viral proto-spacer frequencies show that the viruses fail to lose all three proto-spacers on a single line until just prior to iteration 3800, after the sweep. The host line thus sweeps due to immunity to both viral sub-populations.

Yet, in addition to gradual fixations, model results demonstrate rapid selective sweeps of individual host sub-populations. In order to identify sweeps, we created an algorithm that clusters CRISPR loci into an optimized number of sub-populations in any given iteration (Text S3). To decide on an ‘optimal’ number of clusters in an iteration, we utilized a machine learning cluster validation technique called the ‘silhouette width’ [41]. We then captured iterations in which the predicted number of CRISPR sub-populations precipitously drops to one, indicating a sweep by a member of one ancestral sub-population (Figure 4A). To verify sweeps, we tracked the frequencies of all spacers in a new-end locus position through the period during which the clustering-predicted sweep occurs. Despite competition from numerous other spacers, a single spacer, unique to one diversifying host sub-population (Text S3), rapidly rises to high frequency in this position (Figure 4B). Importantly, the vast majority of virus-host interactions are immune during the sweep period (Figure 5), showing that the rapid loss of host diversity was due to a sweep by a highly immune host rather a bottleneck due to a lack of host fitness.

To understand how a sweep could occur despite model-implemented ‘kill the winner’ dynamics, we reconstructed the strain containing the sweeping spacer identified in Figure 4B. We noticed that the two subsequent spacers added on this strain targeted distinct viral sub-populations, immunizing the host against both dominant viral sub-populations (Figure 5). In this particular case, the viruses were unable to mutate both matching proto-spacers on a single line prior to the host sweep (Figure 5). Thus, while adding spacers that confer immunity to one viral sub-population is common in the model and results in clonal interference among similarly partially immune lines, rapidly acquiring immunity to all viral subpopulations is a rare, ‘multiple mutation’ event [42], which leads to a uniquely immune line that can sweep. More generally, this captures how ‘kill the winner’ cannot maintain spacer diversity in CRISPR loci. Viruses cannot always make the requisite mutations needed to kill a host before that host sweeps. Once trailer-end diversity is lost in even a single rare sweep, trailer-end diversity cannot be regained because distinct spacers are only added at the leader-end.

### Incorporating deletions into the model explains trailer-end memory

While unidirectional spacer addition alone explains the emergence of trailer-end clonality, it does not explain the more basic question of why trailer-end spacers are at all preserved despite rarely matching current viruses (Figs. 1, 2). To probe the potential fitness cost associated with rapidly deleting CRISPR spacers, we introduced random spacer deletions into our *in silico* evolving system. Spacer deletion was implemented by allowing a preset fraction of spacer additions to occur with the loss of a randomly-sized, contiguous spacer block from a random starting point in the locus. A combined add/loss mechanism is consistent with experimental evidence indicating that spacer deletion occurs via homologous recombination [43], [44] and data showing that losses often occur with simultaneous new-end spacer additions [15], [45].

**Figure 6:**
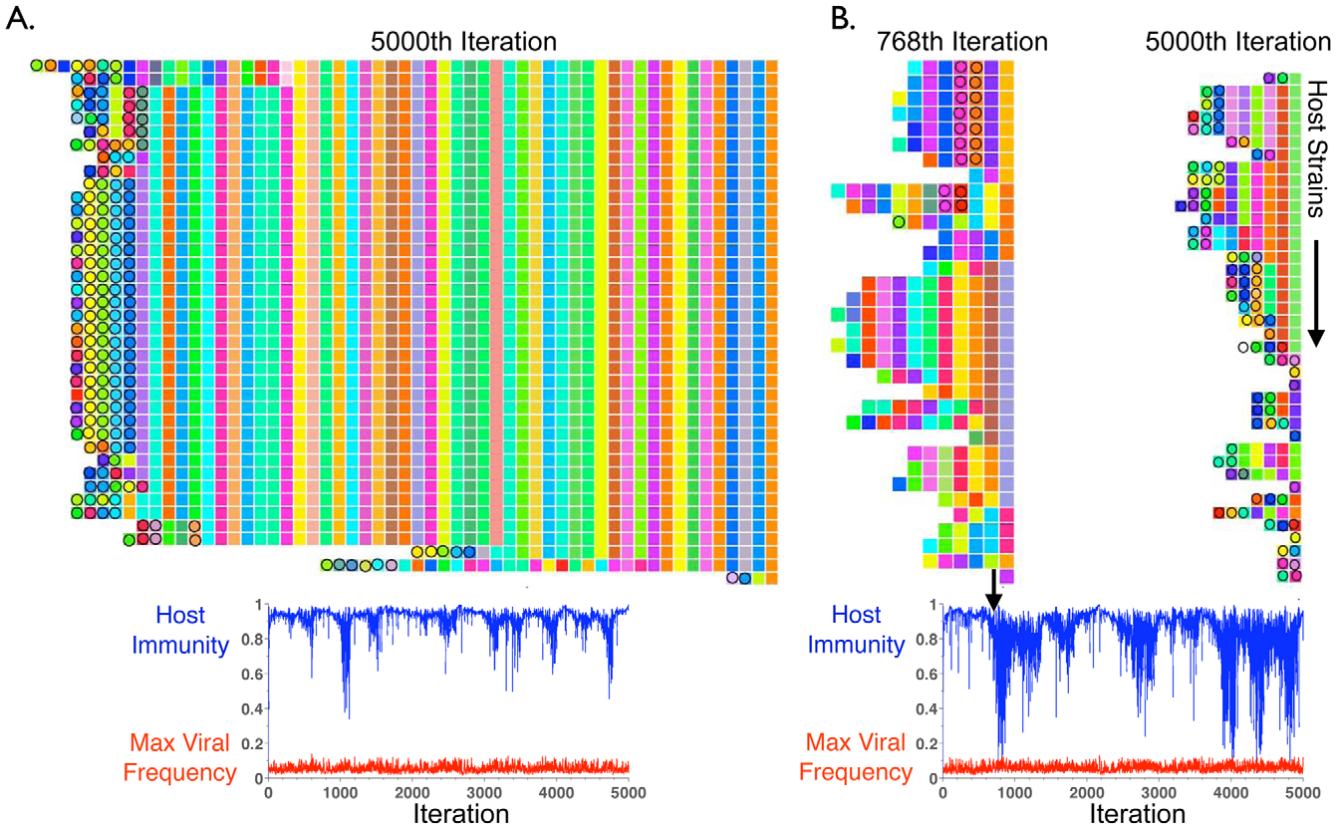
Model shows trailer-end conservation protecting hosts against blooms of old viral sequences. The model is extended to allow a parameterized fraction of (single) spacer additions in host CRISPR loci to occur with deletions of randomly-sized blocks of spacers from random locus positions. The lower panels in (A) and (B) plot host immunity (blue) against maximum viral strain frequency (red) in each iteration. (A) When 5% of additions occur with deletions, trailer-end memory and clonality are preserved. Only new-end spacers target current viruses and CRISPR's antiviral immunity is maintained at high levels across thousands of model iterations. (B) When 50% of spacer additions occur with deletions, trailer-end memory and clonality are purged. Depletions in host immunity occur (lower panel), indicating viral blooms due to the large fraction of interactions in which CRISPR fails to provide immunity (*i.e.*, host and virus do not share spacers). During the predicted bloom at iteration 768, immunity against the top 300 viral strains is conferred by two older spacers, which are lost from most host lines prior to the bloom (Figure S8).

If selection played no role (*i.e.*, spacers conferred no immunity) in CRISPR evolution, the equilibrium number of spacers in a strain's CRISPR locus would roughly be the ratio of spacer addition to loss rates. This is the steady state of the linear differential equation dN/dt = *a*−d*N, where N is the number of spacers, *a*, the spacer addition rate, and, *d*, the spacer deletion rate. Thus, even with selection extending the size of CRISPR loci to maintain spacer immunity, the long-run equilibrium lengths of CRISPR loci should be inversely proportional to their spacer deletion rates. By incorporating the deletion process into our model, we find that when only 5% of spacer additions occur with deletions, CRISPR locus lengths look qualitatively similar to model results with no deletions, with trailer-end conservation and clonality largely preserved (Figure 6A). Conversely, allowing 50% of spacer additions to result in deletions of random spacer blocks purges CRISPR trailer-ends entirely (Figure 6B). Given our experimental data showing that CRISPR loci conserve trailer-ends over time (Figures 1,2), model results predict that the rate of spacer deletion is maintained below a threshold in many natural systems.

To understand why selection would maintain spacer deletion rates below a threshold, we compared the mean fitness of host strains across time under both low and high-loss regimes. Our measure of host mean fitness in an iteration is the fraction of virus-host interactions in which CRISPR provides immunity. While a low-loss rate (5%) produces consistently high levels of host immunity and thus fitness (Figure 6A Lower Panel), dramatic dips in host immunity are observed when the probability of spacer deletion is increased to 50% (Figure 6B Lower Panel). Troughs in host immunity predict rapid viral blooms due to the large number of productive virion producing interactions (Figure S7).

To understand why host immunity is depressed when CRISPR's spacer deletion rate is increased, we reconstructed host CRISPR loci from the time point at which the fraction of immune hosts is at a trough (iteration 768 in Figure 6B). During this predicted viral bloom, the few hosts immune to the top 300 viruses are surprisingly protected by two older spacers (Figure 6B Upper Panel). These older spacers were previously far more prevalent among hosts (Figure S8). Viral proto-spacer mutation eliminated the selection pressure maintaining the two spacers in the hosts, resulting in the rapid loss of the two spacers from most hosts due to the high spacer deletion rate. Viruses managing to preserve the targeted proto-spacers while avoiding extinction could then bloom, free from spacer-driven immunological control (Figure S8).

**Figure 7:**
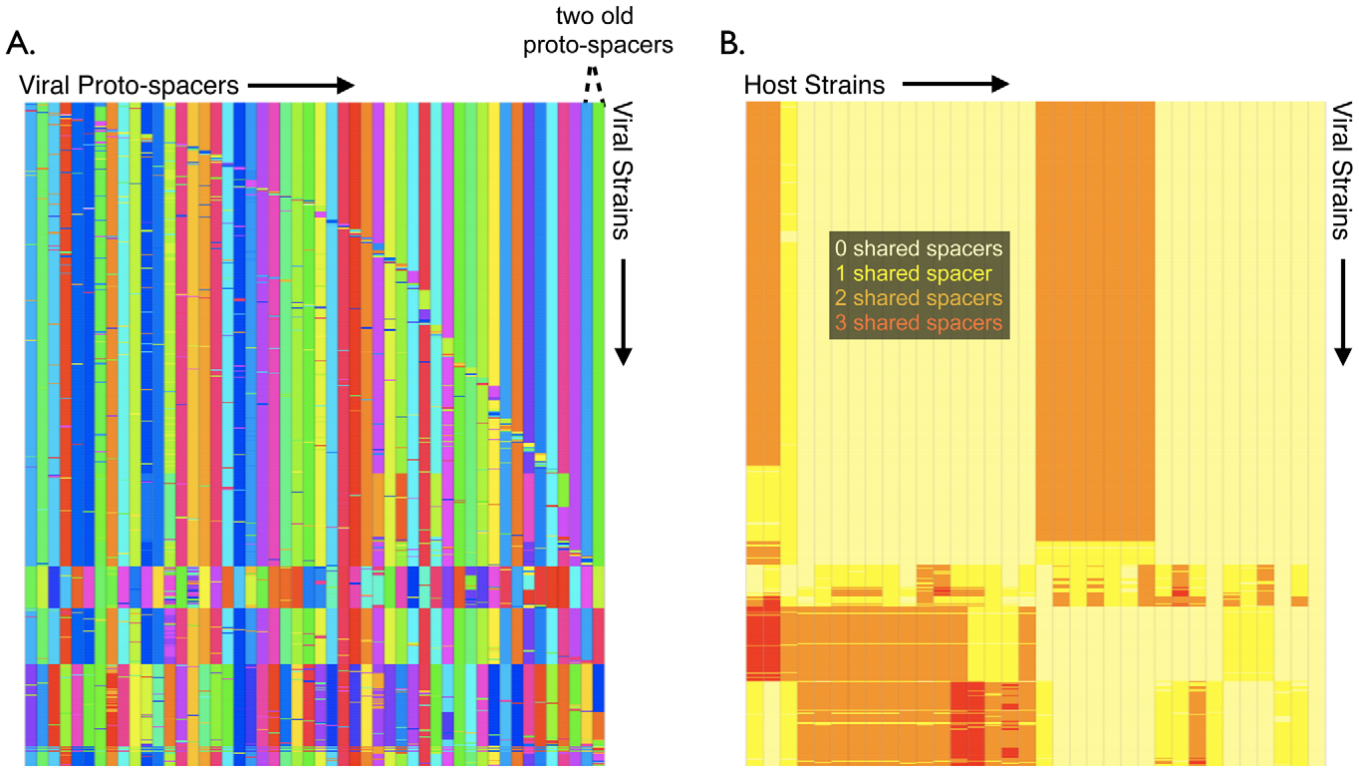
Clustering by immunity reveals diversity during viral bloom. (A) The 651 viruses at the model predicted bloom (iteration 768 in Figure 6B) are shown along the rows, with the virus' 50 aligned protospacers shown along the columns. Distinct proto-spacers are colored differently. Strains are then clustered based on proto-spacer relatedness. The two proto-spacers providing immunity at the bloom (Figures 6B and S8), are shifted to the two rightmost columns for clarity. Five distinct viral sub-populations are observed in the mosaic, with the largest blooming sub-population characterized by closely related mutants sharing the two critical proto-spacers in their rightmost columns. (B) Viral and host sub-populations at the bloom are superimposed on one another to reveal ‘cloud on cloud’ immunity at the bloom. The rows contain viral strains and the columns show host strains. Each entry of the heat-mapped ‘immunity matrix’ shows the number of shared spacers between the respective host and viral strain. Pale yellow color represents no shared spacers (susceptibility), yellow one shared spacer, orange two shared spacers, and red three shared spacers. The silhouette width (Text S3) was maximized to cluster both hosts (columns) and viruses (rows) into an optimal number of sub-populations based on immunity profiles. Distinct host sub-populations possess immunity to distinct viral sub-populations.

Importantly, the viral bloom is not monoclonal: a number of sub-populations can be found within the blooming viral population (Figure 7A). Further, the main viral sub-population, which contains the two older proto-spacers, is rife with new mutants containing polymorphisms in their proto-spacer sequences (Figure 7A). Blooming viral diversity matches the host diversity evident from the CRISPR loci reconstructed during the bloom (Figure 6B). To quantify the correlation between virus and host polyclonalities, we superimposed virus and host strains onto a single matrix, with viral strains allayed along the rows and host strains allayed along the columns (Figure 7B). Each (row, column) entry of the matrix represents the number of shared spacers between the row's viral strain and the column's host strain (*i.e.*, the level of immunity). This results in horizontal immunity vectors for each virus and vertical immunity vectors for each host. We then clustered the viral immunity vectors into an optimal number of viral sub-populations by maximizing the ‘silhouette width’ as above (Text S3) and analogously optimally clustered the column-wise host immunity vectors. Immunity clustering shows a clear pattern of specialization in which distinct host sub-populations coexist through immunity to distinct viral sub-populations in what could be termed ‘cloud on cloud’ immunity (Figure 7B). The presence of distinct immunological niches explains why only seven host strains matched the top 300 viral strains (Figure 6B); the other hosts survived through immunity to less frequent viruses (Figure 7B).

**Figure 8:**
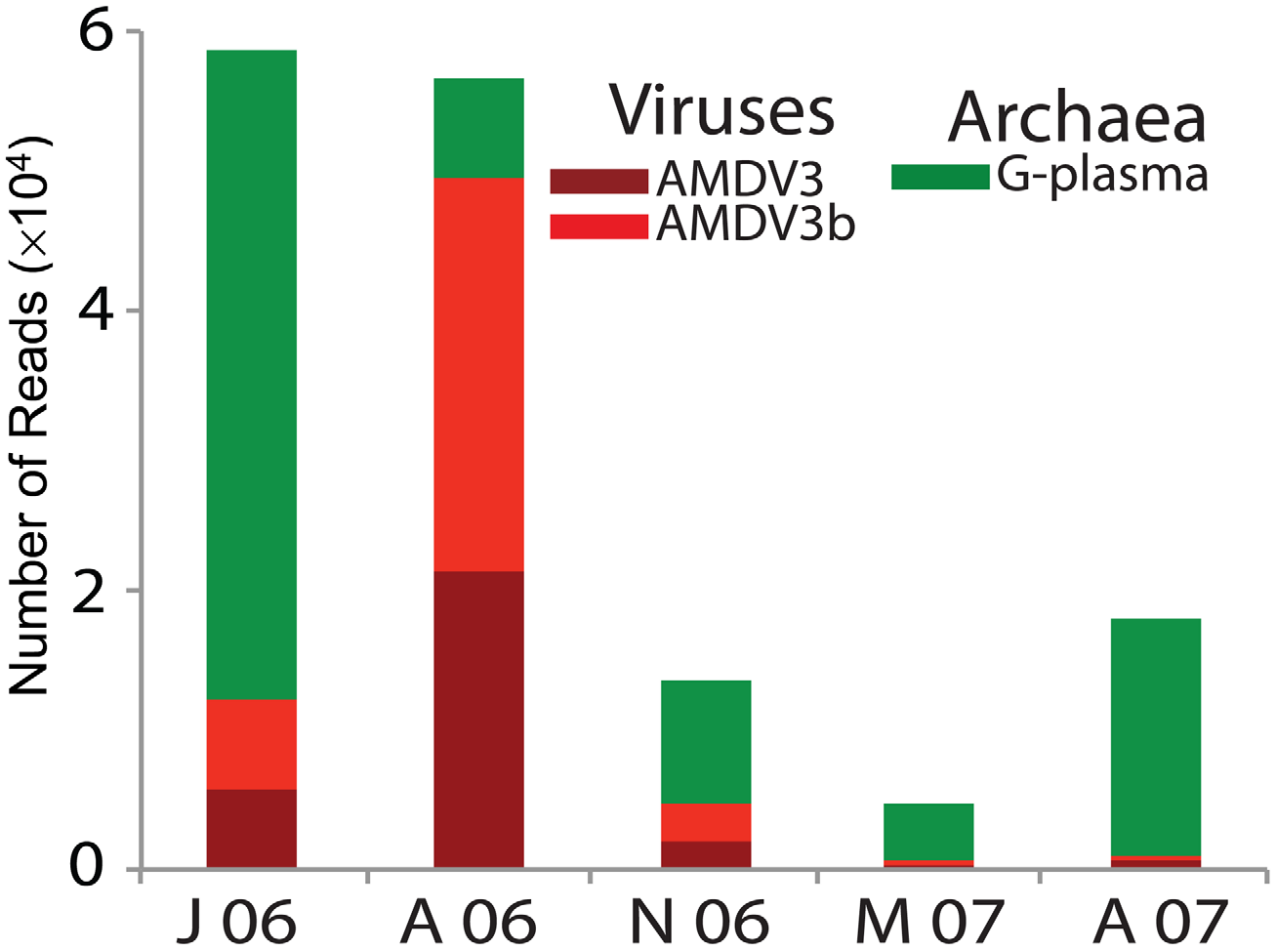
Metagenomic sampling across time captures a natural viral bloom. Number of sequencing reads of G-plasma and its viral populations, AMDV3 and AMDV3b, calculated from the community genomic data at a single location across five time points in 2006–2007. Relative abundances of all archaeal, bacterial, viral and plasmid genomes reconstructed from this community during 2006–2007 are shown in Figure S9. Both Figures 8 and S9, capture a bloom of AMDV3b virus (bright red) at the second time point, August 2006, coincident with the depletion of its archaeal G-plasma host (bright green). Notably, the G-plasma CRISPR loci from these time points were reconstructed in samplings (3)–(7) of Figures 1 and 2. G-plasma contained several spacers exactly matching the blooming AMDV3b sequences prior to the August 2006 bloom (black diamonds in Figure 2).

Matching model predictions of a deletion-induced polyclonal viral bloom, we used the community metagenomic data to capture a viral bloom of AMDV3b despite preexisting spacer immunity its host G-plasma population. We tracked the relative abundances of a number of host and viral species in the AMD consortium through a series of samples collected at a single AMD location between June 2006 and August 2007 (Figures 8 and S9). The G-plasma CRISPR loci from these samplings were shown in Figures 1 and 2 as reconstructions (3)–(7). Relative abundances of host and viral strains were determined by quantifying the number of reads showing high sequence similarity to the reconstructed composite sequences (Methods). While G-plasma was recovered from all samples across time, G-plasma was highly depleted in the August 2006 sampling, coincident with a bloom in the viruses shown to target it, AMDV3 and AMDV3b. Importantly, Figure 2 shows the preexisting presence of trailer-end spacers in G-plasma exactly matching AMDV3b (black diamonds), indicating a putative selective advantage to preserving old spacers and suggesting that spacer deletion between samplings may have driven the rapid proliferation of AMDV3b.

**Figure 9:**
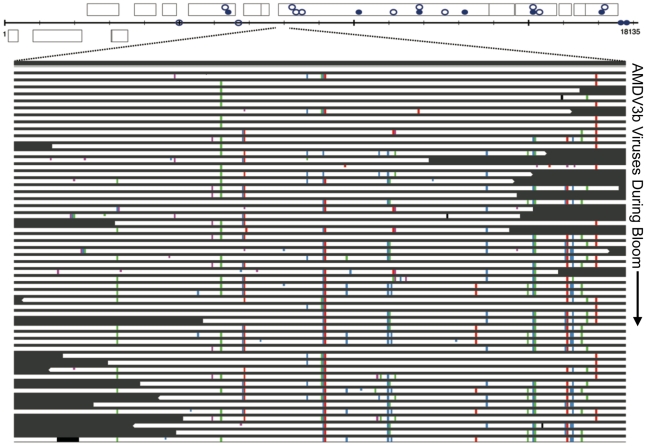
Natural viral bloom is polyclonal. Sequence variation within a gene of the blooming AMDV3b viral population (345 bp field of view). The top bar of the figure represents an 18 kb contig sequence of AMDV3b, with predicted genes shown as boxes. Below the contig, is a close-up view of sequence variation within a single gene. White bars represent aligned sequencing reads, while colored bars indicate SNPs relative to the composite sequence. The black region is a large deleted sequence block in one individual viral genome. Distinct viral sub-populations are captured during the bloom, each sharing common SNPs. Also shown in the figure are regions of the AMDV3b contig that match G-plasma spacers: closed circles in the contig represent perfect matches and open circles represent imperfect matches. When the match between G-plasma spacer and AMDV3b protospacer occurs within a predicted gene, the circle is placed inside the corresponding gene box; matches to intergenic regions are shown below on the contig line.

Further supporting model predictions, the viral bloom is polyclonal with a number of sub-populations clearly recognizable (Figure 9). A monoclonal rather than polyclonal bloom is the expected outcome when viruses out-mutate host immunity (i.e., the successful viral mutant alone blooms), indicating that the bloom was not the result of a recent viral mutation but instead due to CRISPR failing against a wide range of extant viral sequences. Correspondingly, there is no evidence of diminished CRISPR diversity among bloom-surviving G-plasma hosts. In fact, two G-plasma sub-populations, differentiated by distinct trailer-end spacers, precede and survive the crash (Figure 2) as occurs in model simulations in which the deletion rate is high enough to prevent the formation of clonal trailer-ends (Figure 6B).

## Discussion

Here we metagenomically track virus and host populations across time in a natural environment and use a mathematical model to reconstruct the dynamics through which CRISPR loci could evolve between these snapshots. We first capture surprising selective sweeps through which highly diverse CRISPR ‘leader-ends’ become clonal ‘trailer-ends’ across time. Our results also explain why CRISPR loci maintain trailer-end immunities for thousands of microbial generations (immunological memory). Both model and metagenomic data capture blooms of persisting viral sequences against which hosts had preexisting spacer immunity. The model directly shows how accelerated spacer deletions drive these blooms, with precipitous drops in host fitness occurring when spacer deletion is increased. Without viral persistence as a selection pressure favoring memory in CRISPR loci, genomically compact prokaryotes would be expected to purge trailer-end spacers given documented genomic deletion biases and the eventual cost of maintaining excess genomic material [32].

Of course, the genomic cost is likely not significant for each short spacer added. Yet, if CRISPR loci grew without bound, at some point there would be a cost associated to maintaining and transcribing enormous loci. Evidence for a genomic length cost emerges in two recent studies. An elegant analysis noted that highly expressed eukaryotic genes possess significantly shorter introns than less expressed genes, a fact attributed to the ATP-cost of transcribing even short DNA regions [46], [47]. In *Salmonella*, Kuo and Ochman [48] noted that bacterial pseudogenes are deleted faster than they would be by drift alone (which has exponential waiting times), pointing to selection as a driver of genomic compactness [48]. As in the eukaryote study, the few enduring bacterial pseudogenes in [48] appear to be less expressed. Interestingly, elongated CRISPR loci may have an answer to the transcriptional cost problem: they appear to disproportionately produce CRISPR RNA at the leader end [6]. An intriguing possibility is that CRISPR loci could bet-hedge [49], [50], with selection tuning the level of trailer-end spacer transcription to scale with the probability of encountering matching viral sequences.

In pinpointing blooms of persisting viruses as the selection pressure favoring CRISPR memory, we noted a surprising polyclonality in both virus and host in the natural system. Had this been the expected, laboratory-observed bloom in which a virus simply mutates around host immunity [4], [15], the result would have been a monoclonal bloom of the viral variant for which the hosts were not able to acquire spacers in time. For a polyclonal bloom to occur, rather than a single lucky viral mutation, host immunity must fail against a large swath of viruses. There are thus two possibilities for how this polyclonal bloom occurred: either the CRISPR system did not provide any immunity at all (*i.e.*, spacers are not immunogenic), or, as in the model, the hosts prematurely deleted key spacers allowing diversified viruses sharing these key old spacers to resurge and bloom. While we cannot entirely dismiss the first possibility, we did simulate the model under the null hypothesis in which spacers are not immunogenic. In that case, when CRISPR loci evolve neutrally, simulated loci emerge with few spacers and no trailer-end clonality. In contrast, naturally sampled G-plasma loci contain tens of spacers and exhibit dichotomous patterns of trailer-end clonality and new-end diversity. More generally, because the rate of neutral fixation of trailer-end spacers scales inversely with the effective population size [51], large microbial populations make genetic drift an unlikely driver of observed CRISPR locus patterns.

Three previous models have been built to study questions surrounding CRISPR-based immunity. Haerter and colleagues studied how viral diversity is maintained against CRISPR, but their model did not track and reconstruct spacer patterns within CRISPR loci [35]. Levin [34] focused on the fundamental question of why CRISPR loci are found in some but not all microbes, but did not include virus and host mutational processes. It thus could not capture the long-run evolution of CRISPR loci within microbes that do maintain CRISPRs. To model this long-run evolution, He and Deem [33] elegantly applied an HIV-derived differential equation model [52]. Yet, in using an HIV model, He and Deem assumed that CRISPR-immunized Bacteria and Archaea control viral abundances in the same way that cytotoxic CD8^+^ T cells target HIV virions. Thus, viral populations surprisingly decline in their system if all host strains (the viral growth source) are increased by a constant factor, as roughly occurs after an influx of resources. Further, in assuming pre-stipulated locus lengths in which each leader-end spacer addition occurs with a corresponding trailer-end spacer deletion, the model in [52] could not probe whether reducing spacer deletions to increase CRISPR locus lengths is an evolutionarily beneficial strategy.

In protecting against blooms of old viral sequences, model predictions and metagenomic data suggest that CRISPR's immune memory makes it suited for environments in which viruses persist for long periods or remigrate from adjacent regions. CRISPR-based immunity may thus be more prevalent in biofilms than in dilute ocean environments [53]. Immunity against persistent viruses may also explain CRISPR's presence in 90% of sequenced Archaea, which have disproportionately been sampled from extreme environments where viruses tend not to lyse their hosts [13], [54].

More generally, proviral latency is a viral persistence strategy and a clear barrier to eradicating pathogens. A fascinating study recently showed that of the 132 spacers matching viruses in CRISPR loci reconstructed from *Pseudomonas aeruginosa* hospital populations, all spacers matched lysogenic but not lytic viruses [55]. And while these spacers do not appear to block lysogenization, the same group and others have demonstrated CRISPR-mediated control on inserted lysogens, apparently preventing lysogenic induction and infectious spread across susceptible populations such as biofilms [56], [57], [58], [59]. A potential explanation for the demonstrated connection between CRISPRs and lysogenic viruses could be CRISPRs immunological memory. By maintaining old immunities, CRISPR may have evolved to safeguard against reemergences of ancestral viruses from lysogenic dormancies.

## Methods

### Metagenomic sample collection

For the 2006–2007 time series study, biofilms were sampled from the acid mine drainage solution – air interface at the C +75 m location in the Richmond Mine (Iron Mountain, CA - 40°40′38.42″N and 122″31′19.90″W (Elevation ∼3,100′)) in June, August, and November 2006, as well as May and August 2007. Environmental parameters of this site at the times of sampling have been reported previously [60]. Samples were transferred to dry ice on site and stored at −80°C.

### DNA extraction, preparation and sequencing of metagenomic libraries

As described in detail previously [61], for each biofilm collected, high molecular weight DNA was extracted from a 1 g subsample using phenol-chloroform isoamyl. To further remove contaminating extracellular polysaccharides, the DNA was subsequently run on a gel and purified via a QIAquick Gel Extraction Kit (Qiagen, Venlo, Netherlands). Preparation of shotgun metagenomic libraries and pyrosequencing using the 454 Genome Sequencer FLX-Titanium system were performed at the W. M. Keck Center for Comparative and Functional Genomics (University of Illinois, Urbana-Champaign, IL) according to manufacturer's instructions (454 Life Sciences, Branford, CT) [62]. Signal processing and base calling were performed using the bundled 454 Data Analysis Software version 2.0.00.

### Metagenomic data analyses

Sequencing reads from the five libraries were co-assembled using Newbler (GSassembler v. 2.0.01, Roche) using default parameters except for a 95% nucleotide identity and 40 nt minimum overlap requirement. Replicated reads were identified using a previously described protocol based on CD-HIT clustering [63] (>95% identity, >five identical bases at the start of the read, no equal length requirement). Within each CD-HIT cluster, reads that shared the same start position on the assembled contigs were identified and removed except for the longest read. Additional filtering of reads containing ambiguous bases, resulted in a total of 990,386 reads (∼350 Mbp). A second assembly, using identical parameters, was performed using this filtered reads dataset.

For community profiling, read assignment to previously identified genomic sequence bins was performed by blastn analysis (e-value cutoff of e^−20^) using a database of contigs previously assembled and binned from four other Richmond Mine biofilm samples: 5-way, collected in March 2002 [64], [65], UBA and UBA filtrate collected in June 2005 [27], [61], and UBA-BS collected in November 2005 [26].

Contigs representing virus genome fragments were identified based on (a) similarity to previously identified virus contigs recovered from the same system, (b) extreme high depth of sequence coverage (in the case of AMDV3b), (c) assembly curation into genome fragments with detectable sequence similarity to the known viruses, and (in all cases) (d) targeting of the genome sequence fragments by CRISPR spacers. Viruses were determined to replicate in specific hosts based on extensive targeting of their genomes by spacers from host-specific CRISPR loci. Curation of contigs containing reads identified as viral was carried out using Consed [66]. Contigs were then imported into GSMapper and extended manually and joined, where appropriate, so that regions fragmented by elevated sequence divergence could be condensed. Cases of extreme divergence were treated as separate contigs. Locations where genomic datasets were fragmented by gene content differences were noted, and the information used as part of the binning procedure. Viral genomes related to the previously studied AMD viruses but that assembled separately were distinguished. For example, the deeply sampled AMDV3b genome is related to the previously reported AMDV3 population and also to a shallowly sampled AMDV3c population (results not shown) that is also present in the C +75 m dataset.

Strainer [67] was used to visualize single nucleotide polymorphism patterns and other forms of variation. This made use of the “.ace” file generated by GSMapper and read re-mapping step that corrects for homopolymer errors during import into Strainer.

### Processing of sequencing reads for CRISPR analysis

CRISPR spacer analysis was performed on individual sequencing reads rather than contigs generated from an automated assembly. Sanger reads (mate-paired ∼800 bp sequences from each end of an ∼3 kb clone) from the 5-way, UBA, UBA-BS, and UBA filtrate datasets, and 454 reads from the C +75 m series, were used in the reconstruction of both G-plasma CRISPR loci (data are separated by time points in Figures 1 and 2). Any 454 reads containing at least one ambiguous base (“N”) were removed. Using a custom Ruby script, the ends of each 454 read were trimmed until a base passed 20/15 NQS (neighborhood quality standard) [68], with a variation described in [69]. Cross_match (developed by P. Green, University of Washington) was used to remove any remaining B adaptor sequences (from library construction). Phred [70], [71] was used to trim the Sanger sequencing reads and Cross_match was used to filter vector sequence.

### CRISPR data analysis

Sequencing reads that sampled the CRISPR loci were identified based on the presence of specific repeat sequences (see below). Custom Ruby scripts were used to extract CRISPR spacer sequences from 454 and Sanger sequencing reads. We allowed for variation in the repeat sequences to avoid omitting spacer sequences due to errors in sequencing (e.g., homopolymer runs). Spacers were grouped using blastclust (using parameters of 85% identity and 85% length overlap) to remove duplication of groups due to sequencing error. Custom Ruby scripts were used to array CRISPR spacers back onto sequencing reads. Assembly of each locus was manually performed in Microsoft Excel based on overlapping spacer patterns and sampling of the flanking genome on part of the read or its mate pair (in the case of Sanger reads). Where possible, 454 reads were arrayed so that patterns of sequential spacers matched locus regions defined based on Sanger reads. For data presentation in Figs. 2 and 3, unique patterns defined by multiple overlapping 454 reads were condensed to report the longest possible sequence of spacers.

### Detection of spacer matches

Spacer matches were detected using blastn, with parameters for short sequences (G = 2, E = 1, F = F). Perfect matches signify exact matches (100% identity across entire length of spacer) while imperfect matches require at least 85% identity across at least 85% of the spacer. The databases used in the blast searches were composed of AMDV3b sequences recovered in this study. While the database used to detect imperfect matches only contained contig sequences, the database used to detect perfect matches also included the individual sequencing reads that comprised each of the contigs.

### Analysis of community composition in C +75 m time series data

For each individual sample, each read was assigned to a sequence bin (organism or virus type) based on blastn analysis (cutoff<e^−20^). The unassigned category indicates similarity to contigs in the AMD sequence database with unknown affiliation. Note that, as described previously [60], changes in solution pH occurred at the sampling site over the time period studied. This altered the overall community composition, particularly the relative abundances of Bacteria and Archaea.

### Modeling implementation

The mathematical model—see Text S1 for the complete algorithm—was programmed and simulated in MATLAB (version 7.7). Model simulations recorded the spacers in all CRISPR loci across iterations, storing distinct spacers as distinct numbers. Images of CRISPR loci (i.e., spacer patterns) were then produced in R (version 2.11). The R ‘Cluster’ package was used to track the evolution and diversity of CRISPR lineages across time.

## Supporting Information

Figure S1 **Trailer-end conservation and clonality in I-plasma.** Metagenomic reconstructions of the CRISPR loci of an archaeal I-plasma population sampled from the AMD system. As in Figures 1 and 2 of the main text, CRISPR loci, with repeats removed, were reconstructed according to spacer ordering in the metagenomic reads. Identical spacer sequences share the same colored box, except white boxes, which denote cell-specific spacers and black boxes, which show flanking genome. White space indicates unsequenced gaps. When spacers match reconstructed AMDV5 viruses, triangles are inserted (filled triangles show perfect matches, while open triangles show imperfect matches). Notably, all virus-matching spacers occur near the diversifying leader-ends, indicating viral evolution to avoid earlier spacer targeting.
(TIFF)

Figure S2 **Schematic overview of the interaction-based mathematical model.** (**A**) Host strains (rectangles) are defined by spacer content, with virus strains (stars) defined by corresponding proto-spacer sequences. The full mathematical model considers all proto-spacers in defining viral strains, but for ease of display this cartoon only tracks the fitness-impacting viral proto-spacers matching current host spacers. (**B**) Diagram of a representative iteration. Model-stipulated ‘well mixing’ results in dominant host strains being virally challenged more frequently causing negative frequency dependent selection. Thus, the initially frequent host strain (B1) is depleted by the newly dominant viral strain able to productively infect it (V2). Clouds of host and viral strains emerge as viral strains mutate (dotted black lines) and hosts incorporate random new spacers unidirectionally (new colored bars at left ends of hosts). The model is built to predict the patterns of virus-host coevolution that emerge after thousands of iterations.
(TIFF)

Figure S3 **Simulations without emergence period also show trailer-end clonality and sweeps.** In the top panel, we plot the top 50 host strains, by frequency, after 5000 model iterations. The remaining parameters are as in Figure 3, with sufficient host addition to allow for ‘kill the winner’ dynamics. In the bottom panel, host diversity is tracked every 50 iterations across a simulation as in Figure 4A, using the ‘silhouette’ technique to choose an optimal number of clusters per iteration. Note that prior to Iteration 4850, a diversity sweep occurs, implying that sweeps are not artifacts due to the grace period preserving new mutants.
(TIFF)

Figure S4 **Successful lineage in** **Figure 3** **was initially infrequent.** In our model implementation, distinct spacers are represented as distinct numbers, with the exception of 0 which reflects lack of a spacer at a locus position. Each of these nonzero numbers is mapped to a unique color for clarity in the figures. Here we simply identify the successful trailer-end spacer set (highlighted in red) that fixes in Figure 3, noting that initially this lineage was at a low frequency of approximately 0.007.
(TIFF)

Figure S5 **Gradual loss in spacer diversity at one locus position.** Here we track the spacer diversity of the second locus position for the simulation analyzed in Figures 3–5 of the main text. In contrast to the rapid selective sweep observed for the 104th locus column (Figures 4 and 5), the 2nd locus column is characterized by the gradual fixation of one spacer (lineage). Further, despite the presence of negative-frequency dependent selection (‘kill the winner’) in individual model iterations (Figure S2), positive frequency dependent selection is evident across thousands of iterations. This occurs, because host lines of low frequency go extinct throughout the simulation.
(TIFF)

Figure S6 **New-end locus diversifications post-sweep.** In Figure 4A, optimal clustering analysis predicted a selective sweep prior to the 3800th iteration. Yet, by the 4300th iteration, a number of distinct sub-populations were identified by the silhouette-based clustering algorithm. Reconstructions of host loci at 3 representative time points–before the sweep, immediately after the sweep, and 500 iterations after the sweep–show that the clustering analysis correctly predicts new-end diversifications of the sweeping sub-population (i.e., a return of diversity by iteration 4300). A second selective sweep (T = 4800 in Figure 4B) selects for a lineage in one of these sub-populations.
(TIFF)

Figure S7 **Higher loss rate increases likelihood of inferred viral blooms.** Predicted relative abundances for host (blue) and viral (red) populations tracked across iterations. The left panel (low-loss rate regime) shows the predicted relative virus and host abundances for the simulation in Fig. 6A of the main text, while the right panel (high-loss rate regime) shows predicted relative abundances for the simulation in Fig. 6B. Host abundances represent the number of immune host interactions and viral abundances the number of productive interactions multiplied by a laboratory-measured viral burst size of 200 virions per interaction.
(TIFF)

Figure S8 **Predicted viral bloom in high-deletion regime occurs due to host spacer deletions.** In the main text, a nadir in host immunity was shown at the 768th iteration in Figure 6B. Hosts with two key older spacers survived this predicted viral bloom. Here we tracked the frequency of these two spacers through the bloom in both host (top panel) and viral (bottom panel) populations. Spacer 39184 is shown in black and spacer 49611 in red. Note that most hosts lose these two contiguous spacers (Figure 6B) prior to the 740th iteration, when almost all viruses have mutated the corresponding two proto-spacers. Yet, a small remnant viral population maintains these two proto-spacers, proliferating and diversifying against newly non-immune hosts.
(TIFF)

Figure S9 **AMD community composition across time.** The relative abundances of all archaeal, bacterial, plasmid and viral populations are metagenomically reconstructed during the five sampling points in 2006–2007 (corresponding to (3)–(7) in Figures 1 and 2). Each pie represents the total number of reads found in a sample. As in Figure 8, which showed only G-plasma and its viruses, a bloom of AMDV3b virus (bright red) is seen in August 2006 coincident with the depletion of its G-plasma host (bright green).
(TIFF)

Text S1 **Algorithm of mathematical model.** The full algorithm of the CRISPR-virus mathematical model is given in reproducible detail.
(DOC)

Text S2 **Model assumptions.** A description of key model assumptions is provided, with explanations for why each assumption is made and descriptions of how each assumption affects the dynamics of the model.
(DOCX)

Text S3 **Algorithm of optimal clustering routine.** To track host and viral genetic diversity across model iterations, we developed a silhouette-based clustering routine. In a given model iteration, the clustering routine distributes host and viral populations into a chosen ‘optimal’ number of sub-populations. Text S3 describes how this optimal number is chosen and how strains are then divided into their respective sub-populations.
(DOC)
