# Supplementary figures and images for: Persisting Viral Sequences Shape Microbial CRISPR-based Immunity
Source: PLoS Comput Biol. 2012 Apr 19;8(4):e1002475. doi: 10.1371/journal.pcbi.1002475 (PMC3330103; doi:10.1371/journal.pcbi.1002475)

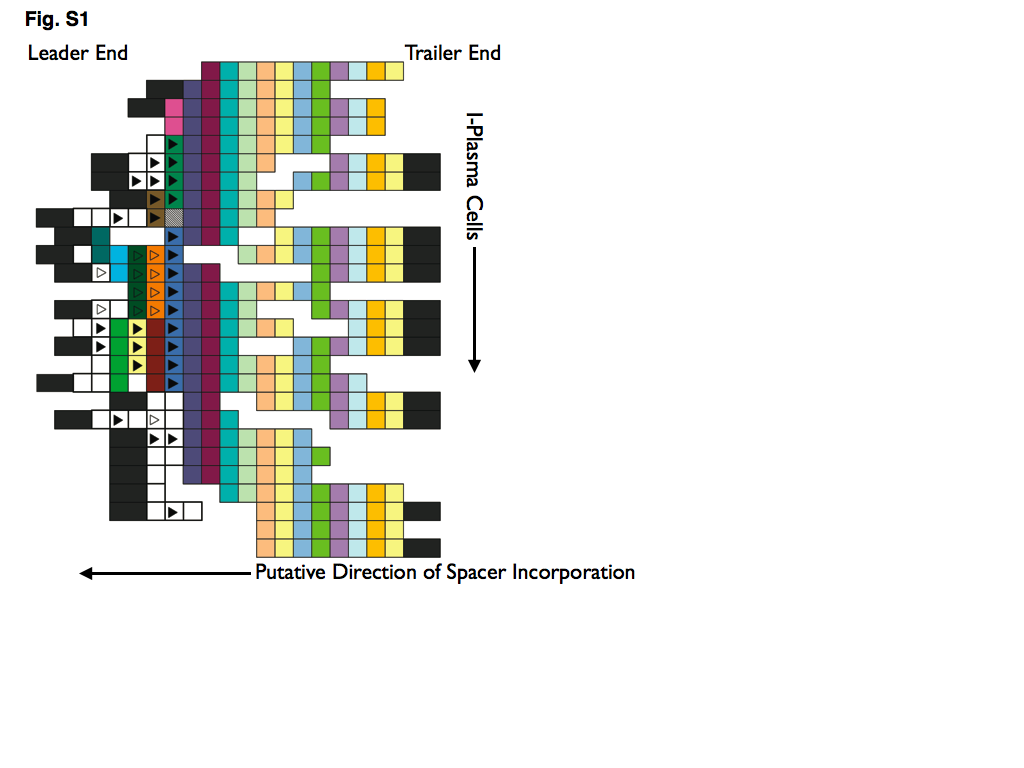

Supplement: Figure S1 — Trailer-end conservation and clonality in I-plasma. Metagenomic reconstructions of the CRISPR loci of an archaeal I-plasma population sampled from the AMD system. As in Figures 1 and 2 of the main text, CRISPR loci, with repeats removed, were reconstructed according to spacer ordering in the metagenomic reads. Identical spacer sequences share the same colored box, except white boxes, which denote cell-specific spacers and black boxes, which show flanking genome. White space indicates unsequenced gaps. When spacers match reconstructed AMDV5 viruses, triangles are inserted (filled triangles show perfect matches, while open triangles show imperfect matches). Notably, all virus-matching spacers occur near the diversifying leader-ends, indicating viral evolution to avoid earlier spacer targeting. (TIFF) [file pcbi.1002475.s001.tiff]

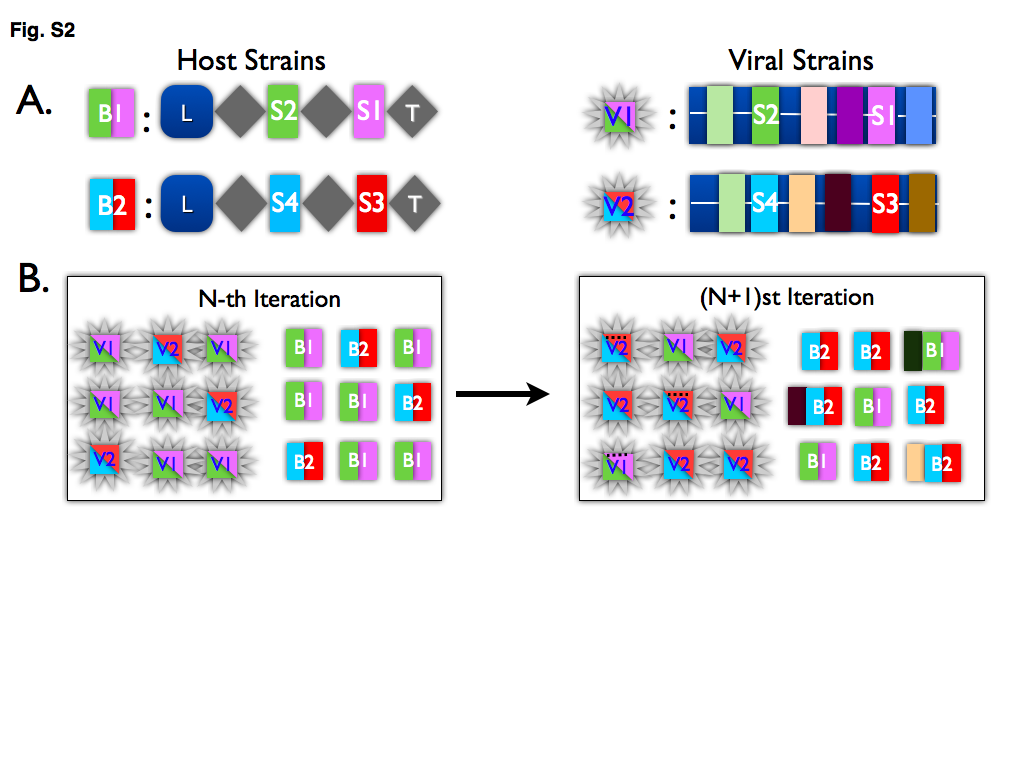

Supplement: Figure S2 — Schematic overview of the interaction-based mathematical model. (A) Host strains (rectangles) are defined by spacer content, with virus strains (stars) defined by corresponding proto-spacer sequences. The full mathematical model considers all proto-spacers in defining viral strains, but for ease of display this cartoon only tracks the fitness-impacting viral proto-spacers matching current host spacers. (B) Diagram of a representative iteration. Model-stipulated ‘well mixing’ results in dominant host strains being virally challenged more frequently causing negative frequency dependent selection. Thus, the initially frequent host strain (B1) is depleted by the newly dominant viral strain able to productively infect it (V2). Clouds of host and viral strains emerge as viral strains mutate (dotted black lines) and hosts incorporate random new spacers unidirectionally (new colored bars at left ends of hosts). The model is built to predict the patterns of virus-host coevolution that emerge after thousands of iterations. (TIFF) [file pcbi.1002475.s002.tiff]

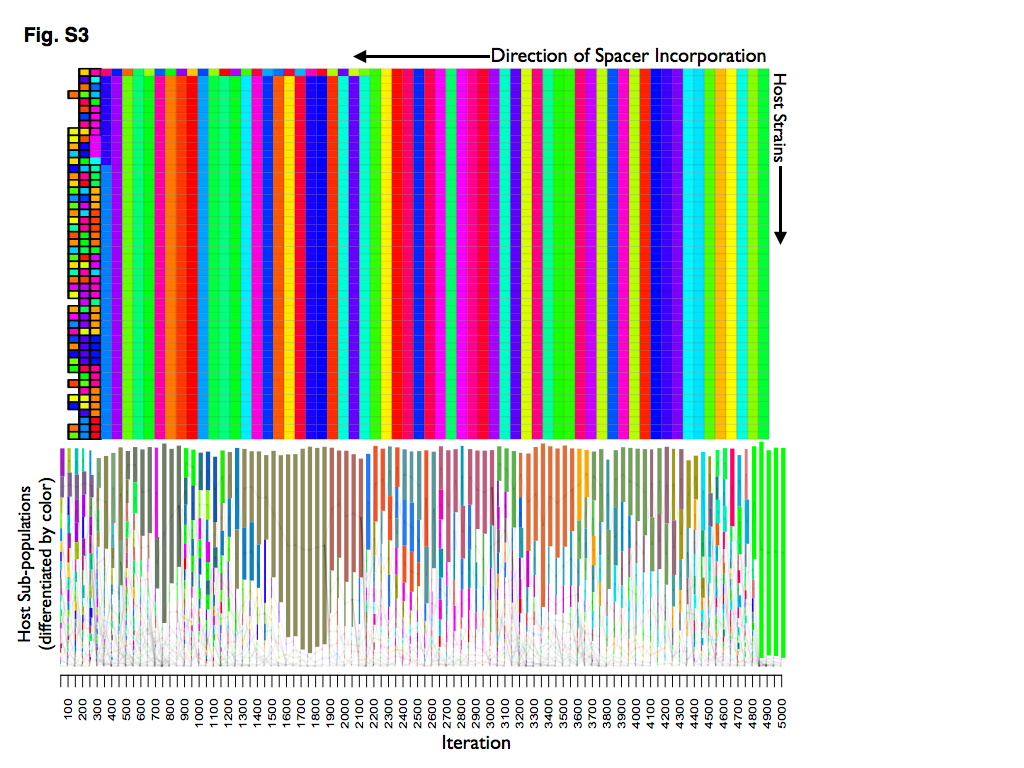

Supplement: Figure S3 — Simulations without emergence period also show trailer-end clonality and sweeps. In the top panel, we plot the top 50 host strains, by frequency, after 5000 model iterations. The remaining parameters are as in Figure 3, with sufficient host addition to allow for ‘kill the winner’ dynamics. In the bottom panel, host diversity is tracked every 50 iterations across a simulation as in Figure 4A, using the ‘silhouette’ technique to choose an optimal number of clusters per iteration. Note that prior to Iteration 4850, a diversity sweep occurs, implying that sweeps are not artifacts due to the grace period preserving new mutants. (TIFF) [file pcbi.1002475.s003.tiff]

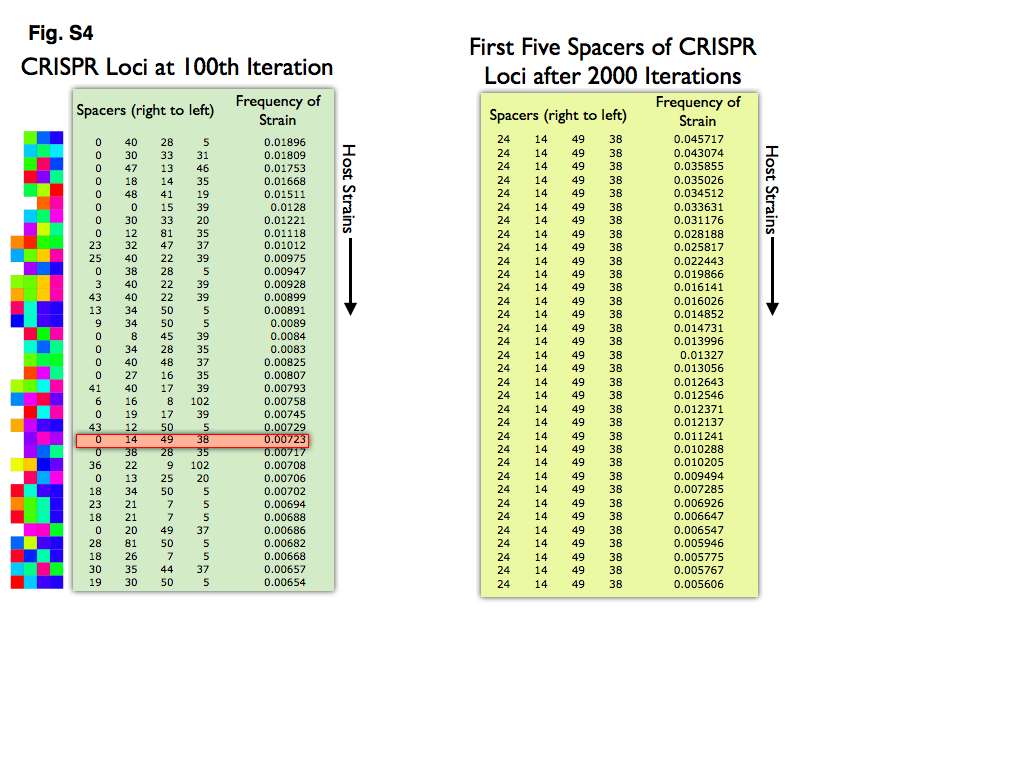

Supplement: Figure S4 — Successful lineage in Figure 3 was initially infrequent. In our model implementation, distinct spacers are represented as distinct numbers, with the exception of 0 which reflects lack of a spacer at a locus position. Each of these nonzero numbers is mapped to a unique color for clarity in the figures. Here we simply identify the successful trailer-end spacer set (highlighted in red) that fixes in Figure 3, noting that initially this lineage was at a low frequency of approximately 0.007. (TIFF) [file pcbi.1002475.s004.tiff]

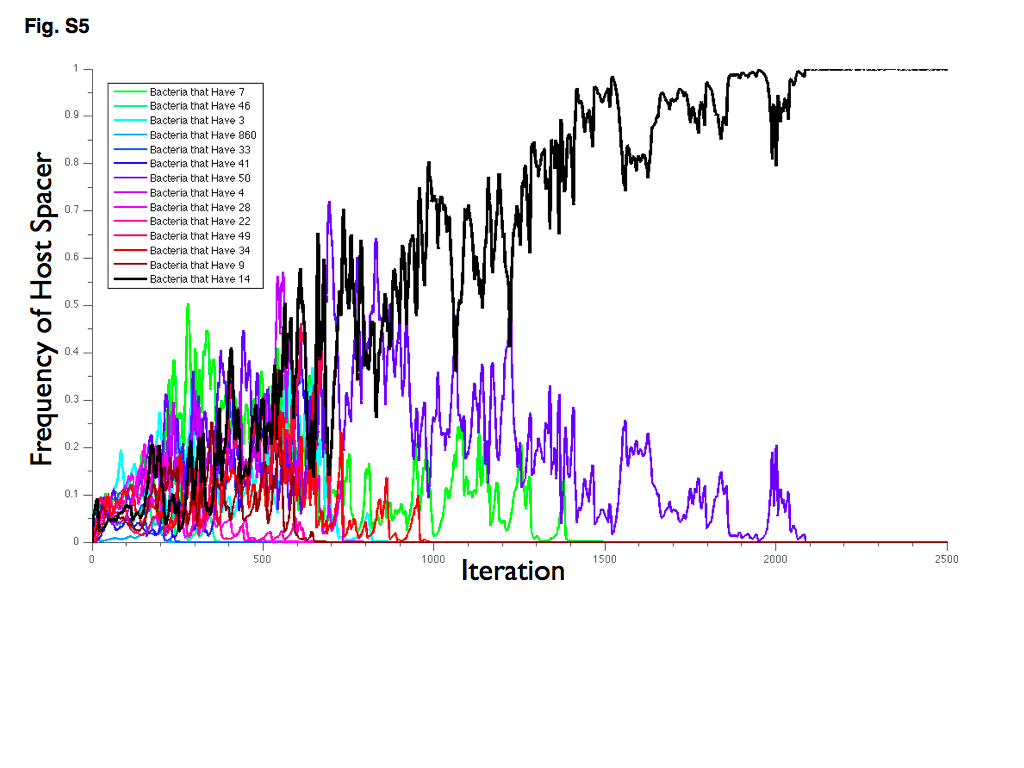

Supplement: Figure S5 — Gradual loss in spacer diversity at one locus position. Here we track the spacer diversity of the second locus position for the simulation analyzed in Figures 3– 5 of the main text. In contrast to the rapid selective sweep observed for the 104th locus column (Figures 4 and 5), the 2nd locus column is characterized by the gradual fixation of one spacer (lineage). Further, despite the presence of negative-frequency dependent selection (‘kill the winner’) in individual model iterations (Figure S2), positive frequency dependent selection is evident across thousands of iterations. This occurs, because host lines of low frequency go extinct throughout the simulation. (TIFF) [file pcbi.1002475.s005.tiff]

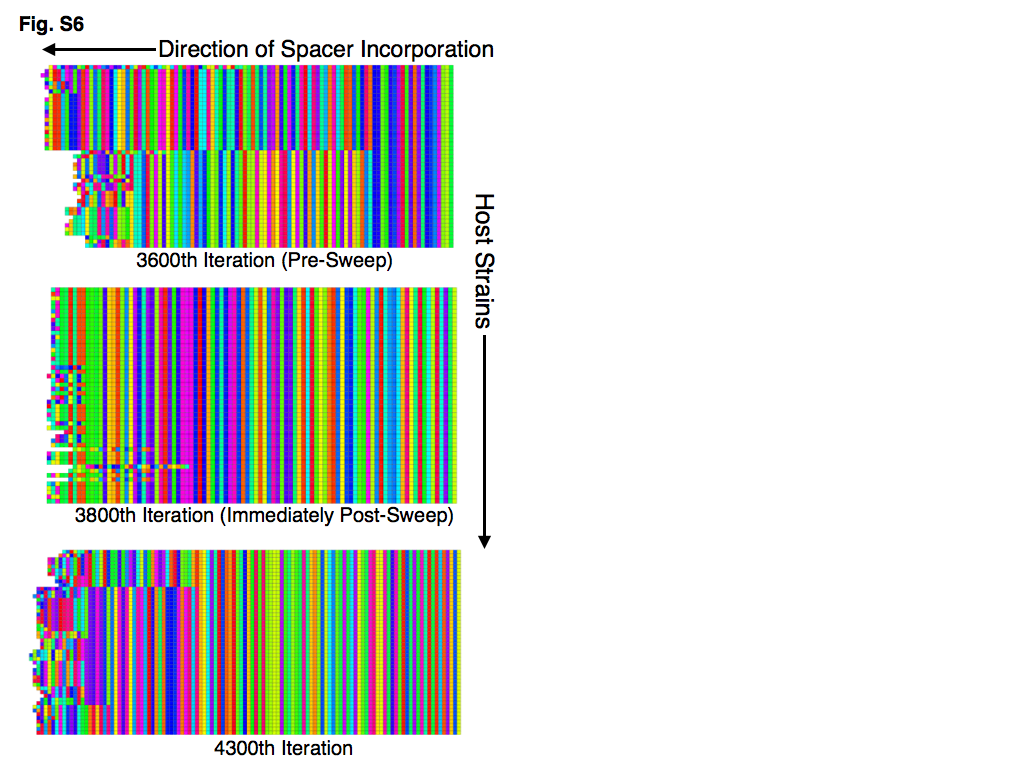

Supplement: Figure S6 — New-end locus diversifications post-sweep. In Figure 4A, optimal clustering analysis predicted a selective sweep prior to the 3800th iteration. Yet, by the 4300th iteration, a number of distinct sub-populations were identified by the silhouette-based clustering algorithm. Reconstructions of host loci at 3 representative time points–before the sweep, immediately after the sweep, and 500 iterations after the sweep–show that the clustering analysis correctly predicts new-end diversifications of the sweeping sub-population (i.e., a return of diversity by iteration 4300). A second selective sweep (T = 4800 in Figure 4B) selects for a lineage in one of these sub-populations. (TIFF) [file pcbi.1002475.s006.tiff]

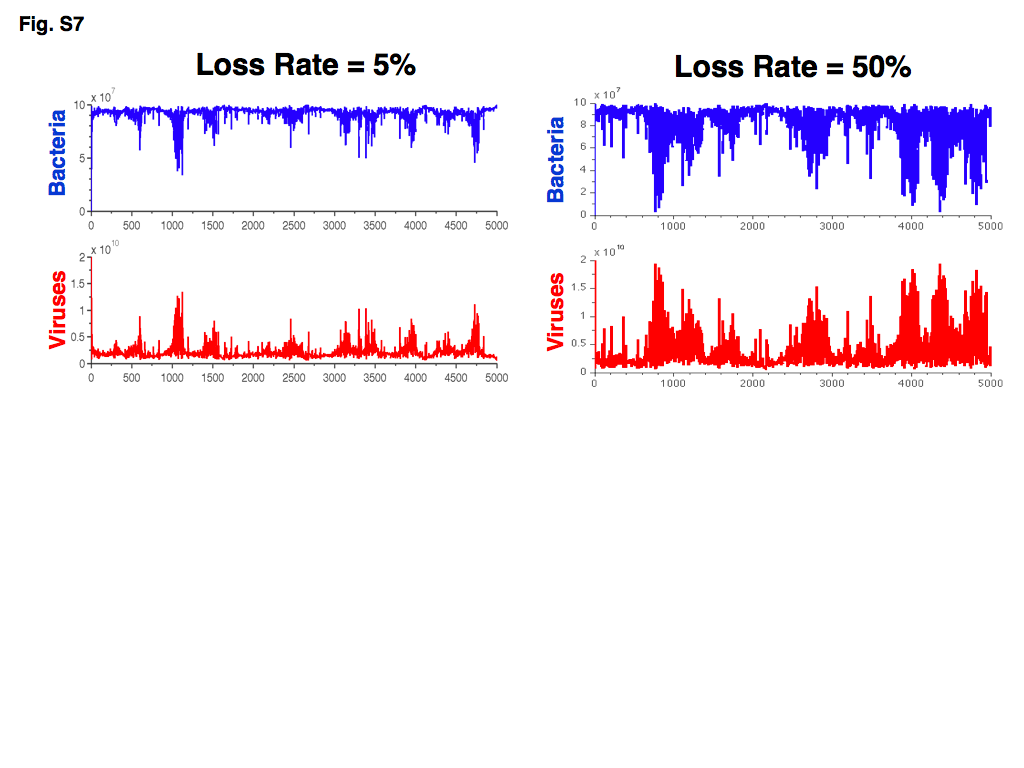

Supplement: Figure S7 — Higher loss rate increases likelihood of inferred viral blooms. Predicted relative abundances for host (blue) and viral (red) populations tracked across iterations. The left panel (low-loss rate regime) shows the predicted relative virus and host abundances for the simulation in Fig. 6A of the main text, while the right panel (high-loss rate regime) shows predicted relative abundances for the simulation in Fig. 6B. Host abundances represent the number of immune host interactions and viral abundances the number of productive interactions multiplied by a laboratory-measured viral burst size of 200 virions per interaction. (TIFF) [file pcbi.1002475.s007.tiff]

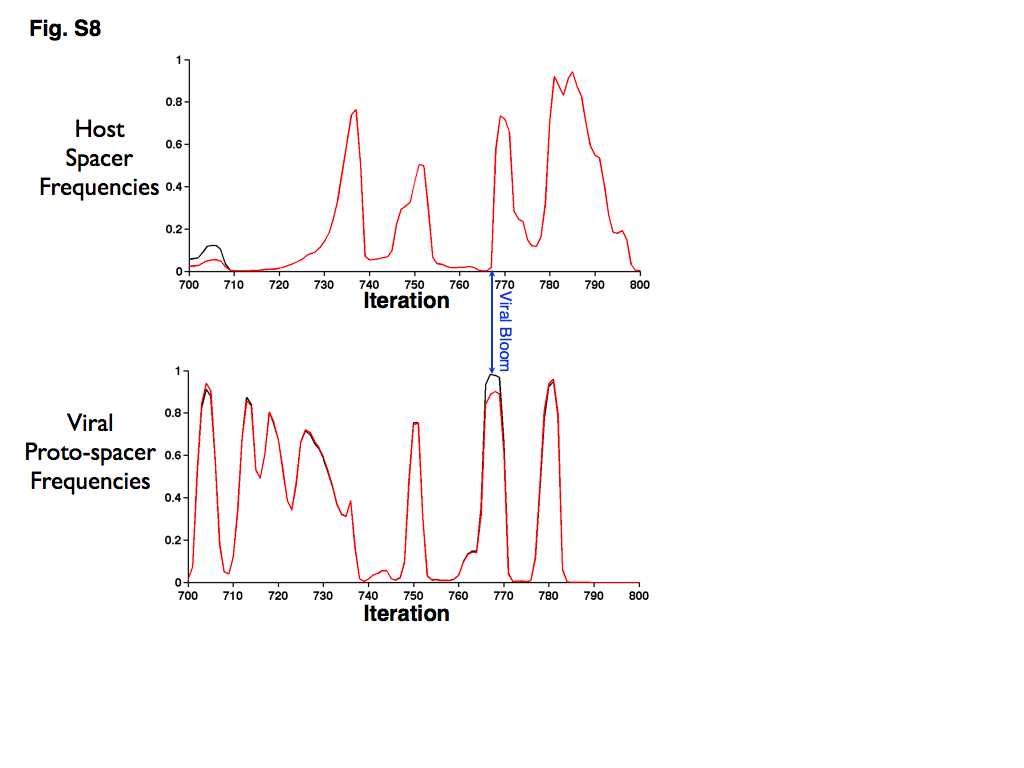

Supplement: Figure S8 — Predicted viral bloom in high-deletion regime occurs due to host spacer deletions. In the main text, a nadir in host immunity was shown at the 768th iteration in Figure 6B. Hosts with two key older spacers survived this predicted viral bloom. Here we tracked the frequency of these two spacers through the bloom in both host (top panel) and viral (bottom panel) populations. Spacer 39184 is shown in black and spacer 49611 in red. Note that most hosts lose these two contiguous spacers (Figure 6B) prior to the 740th iteration, when almost all viruses have mutated the corresponding two proto-spacers. Yet, a small remnant viral population maintains these two proto-spacers, proliferating and diversifying against newly non-immune hosts. (TIFF) [file pcbi.1002475.s008.tiff]

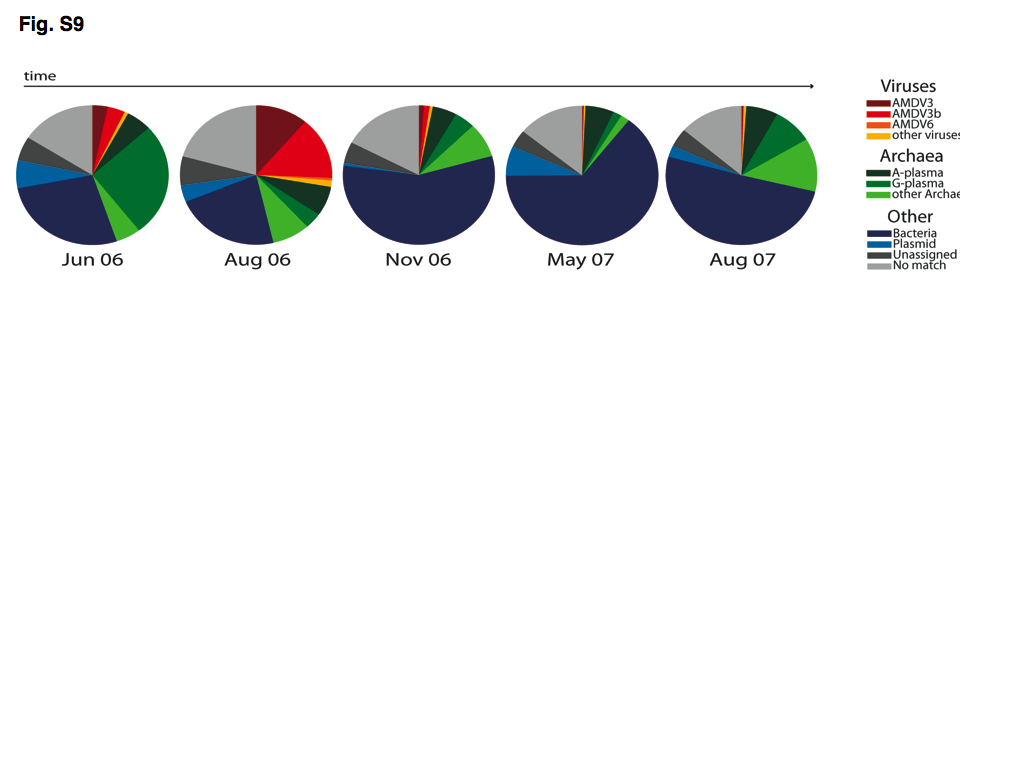

Supplement: Figure S9 — AMD community composition across time. The relative abundances of all archaeal, bacterial, plasmid and viral populations are metagenomically reconstructed during the five sampling points in 2006–2007 (corresponding to (3)–(7) in Figures 1 and 2). Each pie represents the total number of reads found in a sample. As in Figure 8, which showed only G-plasma and its viruses, a bloom of AMDV3b virus (bright red) is seen in August 2006 coincident with the depletion of its G-plasma host (bright green). (TIFF) [file pcbi.1002475.s009.tiff]
