# Supplementary material for: Persisting Viral Sequences Shape Microbial CRISPR-based Immunity
Source: PLoS Comput Biol. 2012 Apr 19;8(4):e1002475. doi: 10.1371/journal.pcbi.1002475 (PMC3330103; doi:10.1371/journal.pcbi.1002475)
Supplement: Text S1 — Algorithm of mathematical model. The full algorithm of the CRISPR-virus mathematical model is given in reproducible detail. (DOC) [file pcbi.1002475.s010.doc]

**Text S1 Weinberger et al.,**

**Model Algorithm**

**Summary of Model**

To simulate the long-term coevolution of CRISPR loci and CRISPR-targeted viruses in natural communities, we designed a simple, frequency-based model in which mutating viruses target spacer-incorporating CRISPR^+^ hosts. Host and viral populations are both divided into strains. Hosts with fully identical spacers are grouped into a single host strain and viruses with identical proto-spacers are grouped into a single viral strain. We then tracked by frequency and genotype across discrete, non-overlapping iterations.

In each iteration, large numbers of host and viral strains interact. We classify all virus-host interactions are either ‘productive’ or ‘immune.’ In a productive interaction, the lytic virus successfully kills the host. Conversely, in an immune interaction the host survives the interaction and the virus is destroyed. The model implements natural selection in each iteration by simply resetting host and viral frequencies to be the respective fractions of immune (host) and productive (virus) interactions. Thus, selection increases the frequencies of host strains with broadly immunogenic spacers, and analogously increases the frequency of viral strains lacking proto-spacers found in the dominant host lines. Affecting selection in the subsequent model iteration, new mutants emerge during virus-host interactions, as some hosts unidirectionally incorporate viral proto-spacers and some viruses mutate proto-spacers. In addition to creating new mutants, at the end of each iteration, the model clears older host and viral strains, which have drop below a parameterized minimum frequency.

**Initialization**

The model is generally initialized with one non-immune host strain having no spacers and one viral strain possessing proto-spacers 1 through *S*. This initial condition was relaxed, with no substantive effect on the long-term dynamics. For example, the simulation shown in Figure 2 began with two distinct immune host lines.

**Model Iteration**

*Step 1: Host and Viral Strains Interact According to Well-Mixing.* The preset number of virus-host interactions in each iteration, *k* (Table 1 of main text)*,* is divided among all pairs of host and viral strains according to well-mixing (mass-action). Mathematically, for each viral strain (**V*_i_***) and each host strain **(B*_j_*)**, the number of interactions between **V*_i_*** and **B*_j_*** in the *t^th^* iteration is:

**[Interactions(***t***)]**_ij_ =*k**(Freq(**V*_i_***(*t*-1)))*(Freq(**B*_j_***(t-1)))

Note that Freq(**V*_i_***(*t*-1)) and Freq(**B*_j_***(*t*-1)) are the frequencies of **V_i_** and **B_j_** at the end of the previous iteration. When *t*=1, the initializing frequencies are used.

*Step 2: New Strain Frequencies Determined by Fitness in Last Iteration.* For each strain-pair **(V_i_, B_j_)** in the given iteration, the number of spacers shared by the virus-host strain pair is counted. *f* is the parameterized function deciding what fraction of strain-pair interactions is productive given this number of shared spacers. Given experimental data showing that more shared spacers between a virus and host makes their interactions less likely to be productive [1,2], *f* is set as a monotonically decreasing function of the number of shared spacers (Table 1). Thus, the number of productive interactions for each  **(V_i_, B_j_)** strain-pair in the *t^th^* iteration is:

**[Productive Interactions**(*t)***]***_ij_* = [***f***(**[Immunity**(*t*)**]*_ij_***)]•[**Interactions**(*t*)]*_ij_*

As an interaction is either productive or immune in the model, the matrix of **Immune Interactions** during iteration t is:

**Immune Interactions**(*t*) = **Interactions**(*t*) **- Productive Interactions**(*t*)

To determine the current frequencies of virus and host, we note that a *viral* strain’s frequency in the current iteration should depend on its ability to have *productively infected* hosts during the previous generation. Conversely, a *host* strain’s frequency in the current generation should depend on its ability to have been *immune* to viral infections during the previous generation. Thus, the model determines current viral and host strain frequencies to be (the prime symbol denotes the fact that these frequencies will change as mutants arise):

*Step 3: Unidirectional Host Spacer Addition.* For each host strain **B_j_**, the number of spacer-adding mutant strains **N_Bj_(t)** comes from the following formula, where P*B_add* is the probability that a host strain adds a spacer during any interaction.


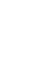


Importantly, Step 2 shows that a host strain’s frequency increases (monotonically) with its probability of being immune to a random viral strain. Thus, by making the number of spacer addition progeny proportional to a host strain’s frequency, this equation assumes that immune strains are more likely to add spacers than non-immune strains. This is consistent with experimental data showing that non-immune hosts have little opportunity to add spacers before productive viral infection overwhelms the cell [1].

For strains in which **N_Bj_(t)**>=1, the model creates all **N_Bj_(t)** mutant strains independently. Further, the particular spacer unidirectionally added by each of these lines is chosen randomly from the distribution of viral proto-spacers in that iteration. We choose directly from proto-spacer distributions, because the well-mixing hypothesis ensures that the relative frequencies of proto-spacers in the viral population matches the relative frequencies of the proto-spacers in the viral strains that each host strain interacts with.

The mutant, like all mutants in the model, is then initialized at a small fraction of its parent’s frequency (Table 1 of main text). This is because mutant and parent are almost identical in spacer sets and thus in immune profiles and fitness (and analogously for viral mutants and parents who share infectivity profiles). Long iterations then offer mutants substantial numbers of interactions to approach sizable fractions of their parent’s frequencies (Text S2).

Finally, each new mutant strain is given an individualized random emergence period in which it avoids the model’s clearance of low frequency strains. We ran the model without the emergence period to assure that results were not artifacts on this assumption (Figure S3).

*Step 3b: Random Spacer-Adding Mutants Lose Blocks of Spacers.* To probe whether deletions of trailer-end spacers depress host fitness by compromising host immunity, we allowed a tunable expected fraction of spacer adding mutants to delete random blocks of spacers from random locus starting points during the addition process.

*Step 3c: Renormalization of Strain Frequencies.* The frequencies of all host strains are renormalized to sum to 1. Consistent with our above well-mixing assumption, a population-wide renormalization assumes that all resident strains suffer equally from the emergence of new mutants.

*Step 4: Mutation of Viral Proto-spacers.* For each viral strain **V_i_**, the resulting number **N_Vi_(t)** of mutant strains that each mutate a proto-spacer during the interactions of the *t^th^* iteration is:

P*v_mut* represents the probability that a virus mutates a proto-spacer in an interaction. Analogous to the hosts, the viral mutation equation makes viral proto-spacer mutations most likely to occur in the viral strains of highest frequency. Thus, proto-spacer mutation generally occurs subsequent to productive infections.

If **N_Vi_(t)**>=1, we create the **N_Vi_(t)** mutant lines of **V_i_** independently. For each of these strains, the particular proto-spacer mutated is chosen randomly from the set of proto-spacers possessed by **V**_i_. Because proto-spaces are over 30 base-pairs long and even single base-pair proto-spacer mutants have been shown to prevent CRISPR immunity in some cases [1,2], we made an ‘infinite allele’ [5] assumption and presumed each proto-spacer mutant unique (*i.e.*, no back-mutation).

Finally, the frequencies of all viral strains are renormalized to sum to 1, consistent with the well-mixing assumption.

*Step 5: Clear Lowest Frequency Strains and Take Metagenomic Snapshots.* Given that unlimited numbers of unfit strains cannot persist at low-frequencies indefinitely, the model clears the least frequent viral or host strains, excepting new mutants in their emergence iterations. For both host and viral populations, the model collects the surviving strains, including emergence-period strains, and renormalizes frequencies so that they sum to one in host and virus populations, respectively. For each surviving **B_j_** and each surviving **V_i_,** the frequencies at the end of the *t-th* iteration are defined to be Freq(**B_j_(t))** and Freq(**V_i_(t))**, respectively. The model takes a snapshot of all surviving host and viral strains, capturing both spacer content and relative strain frequencies. In all but the final iteration, the model returns to step 1.

**References**

1. Barrangou R, Fremaux C, Deveau H, Richards M, Boyaval P, et al. (2007) CRISPR provides acquired resistance against viruses in prokaryotes. Science 315: 1709-1712.

2. Deveau H, Barrangou R, Garneau JE, Labonte J, Fremaux C, et al. (2008) Phage response to CRISPR-encoded resistance in Streptococcus thermophilus. J Bacteriol 190: 1390-1400.

3. Garneau JE, Dupuis ME, Villion M, Romero DA, Barrangou R, et al. (2011) The CRISPR/Cas bacterial immune system cleaves bacteriophage and plasmid DNA. Nature 468: 67-71.

4. Drake JW, Charlesworth B, Charlesworth D, Crow JF (1998) Rates of spontaneous mutation. Genetics 148: 1667-1686.

5. Kimura M, Crow JF (1964) The Number of Alleles That Can Be Maintained in a Finite Population. Genetics 49: 725-738.
