# Supplementary material for: Persisting Viral Sequences Shape Microbial CRISPR-based Immunity
Source: PLoS Comput Biol. 2012 Apr 19;8(4):e1002475. doi: 10.1371/journal.pcbi.1002475 (PMC3330103; doi:10.1371/journal.pcbi.1002475)
Supplement: Text S2 — Model assumptions. A description of key model assumptions is provided, with explanations for why each assumption is made and descriptions of how each assumption affects the dynamics of the model. (DOCX) [file pcbi.1002475.s011.docx]

**Text S2 Weinberger et al.,**

Detailed Justification of Model Assumptions

Like any model of a complex natural system, our mathematical model of CRISPR-virus coevolution necessitated simplifying assumptions. In the main text, a number of these assumptions were briefly probed: here we describe them in detail and offer support for each. We further amend the model to demonstrate that our conclusions are robust to several simplifications applied.

**i) Virus and Host Populations Coexist at Large Abundances (i.e., 10^6^ interactions/iteration)**

Empirical support for the long-run coexistence of host and virus in natural microbial systems comes from two metagenomic studies in distinct environments. In the first study, Rodriguez-Brito et al., tracked consistently high loads of virus and host in four aquatic regions across a year-long period[1]. Similarly, in our study, we reconstructed the relative abundances of archaeal CRISPR loci and viruses in an acid mine drainage system across the last two years of our six-year metagenomic time series experiment (Fig. 3c). In each sampling, we recovered both host and viral genomes, implying that even if local extinctions occur, the populations are re-seeded from elsewhere in the system prior to the following sampling.

With host and virus continually extant in this system, one can wait long enough such that any preset number of interactions occurs in an iteration. We thus define a model iteration as the variable-duration period in which a parameterized number of interactions occurs. Generally 10^6^ was utilized as the number of interactions per iteration, but we modified this value to probe model robustness. Importantly, the number of interactions per iteration only affects the number of mutants that arise per iteration; it does not directly affect a strain’s relative fitness and thus its frequency. In a seminal study on mutation rates across species, Drake et al., note that the per-genome mutation rate of DNA viruses is consistently approximately 3•10^-3^ mutations per replication [2]. That is, independent of genome length, 10^6^ interactions allows viruses approximately 3•10^3^ mutants per generation. Given that viral proto-spacers are defined as regions immediately preceding a specific 2-3 base-pair nucleotide motifs (PAMs), a back of the envelope estimate argues that 1/16^th^ to 1/64^th^ of viral genomes is proto-spacer material. So 10^6^ interactions yields on the order of 100 proto-spacer mutations per iteration, as parameterized in the model (Table 1). With approximately 100 viral proto-spacer mutations in each iteration, the model assures that the majority of the 50 viral proto-spacer loci in our simulations have a chance to evolve.

An advantage of nondimensionalizing the model in terms of time is that interaction-defined iterations no longer require parameterizations of unknown, system-dependent rates. For example, the average rate at which viral particles contact and infect host cells is poorly estimated even in the well-studied case of HIV in a closed human host; in an open, unstudied natural setting one would have trouble choosing its value. Interaction-based iterations also appropriately define host and virus mutation ‘rates’ as the respective probabilities in which each virus-host interaction results in a host spacer addition or a viral proto-spacer mutation. These probabilities are driven by the basic genetic architectures of host and phage and should thus be greatly system-independent, as is indicated by the relatively constant per-genome mutation rates of DNA microbes [2].

**ii) Virus and Host Strains Interact According to Exact Frequencies**

We excluded the effects of random sampling in the interaction stage of the initial model for two reasons: a) strong selection has been experimentally demonstrated in CRISPR-virus interactions and b) empirical microbial systems consist of large numbers of host and virus. Importantly, while ignoring sampling noise in choosing the numbers of each host and viral strain that interact, we did consider a related ‘demographic stochasticity’ in the mutation stage of the model, choosing the number of mutants of each host and viral parent strain from a Poisson Distribution.

The *Law of Large Numbers* guarantees that as a population grows large, the relative frequency at which a particular event is sampled approaches the true probability of the event in question, minimizing the impact of genetic drift. Given the stably high number of viruses and hosts repeatedly sampled in the Rodriguez-Brito study[1], the total population sizes of viruses and hosts are kept large in our model. Thus, any host or viral strain with a non-negligible frequency has an abundance far from zero and is not susceptible to drift-driven extinctions. Hosts with low frequencies (less than 10^-6^) are cleared from the model, with the exception of new mutants, which we now consider.

Among new mutants, one would ordinarily need to include drift because mutants should arise at frequency 1/N (where N is the respective host or viral population size at that time). With initially low frequencies prior to ‘invading’, new mutants are susceptible to drift-driven extinctions. In our model, however, new mutants are initialized at moderate frequencies based on their parent fitnesses, allowing us to ignore sampling noise. As we explain below in the section on mutant frequency initialization, we do this because of the extremely strong selection measured in CRISPR-virus interactions[3,4]. Population genetics shows that the probability of a new mutant avoiding extinction due to drift (i.e., ‘establishing’) in a large population is solely a function of its selection advantage [5].

**iii) Strains Are Well-Mixed (mass-action)**

The model assumes that wherever one looks across an environment the probability of encountering a particular host or viral strain—conditional on encountering some host or viral strain, respectively—is uniform. We thus assume that a strain’s frequency in an iteration gives the conditional probability that the strain is observed at any point in the environment.strain-specificspatial reservoirs in which viruses and hosts of particular types are clustered.

Our model ignores spatial heterogeneity for three reasons. First, we built a first-pass general model for natural systems whose ecologies are unknown and whose topographies can vary. Thus, we have no *a priori* knowledge of the system-specific spatial heterogeneities to incoporate into the model. Finally, spatial heterogeneity would only strengthen the major result of this paper—i.e., that old-end spacers are conserved against rare resurgences of low-abundance viruses—because spatial heterogeneity (reservoirs) is a primary mechanism for viral persistence [6,7].

**iv) No Back-Mutation (Infinite Alleles Model)**

With more than 30 base-pairs per proto-spacer, viruses possess innumerable degrees of freedom in mutating proto-spacers mutate around CRISPR-based immunity. In fact, single base-pair mutations in either the proto-spacer or flanking PAM (proto-spacer adjacent motif) region have been shown to in some cases prevent CRISPR-mediated immunity [3,4,8]. With so many mutational escape routes available, including many synonymous mutations, the probability of a strain mutating back to an antecedent becomes negligible. Thus, as in Kimura’s well-known Infinite Alleles Model [9], we assume that each viral mutant arises from a bin of limitless allele types, choosing a unique, unseen proto-spacer during each viral mutation. Incidentally, this assumption also strengthens the main result of blooms of previously targeted viral sequences, showing that they are not built-in to the model via back-mutation.

**v) Hosts Add and Delete Spacers, but Do Not Mutate Spacers**

While host CRISPR loci add and delete spacers in the model, we do not consider the mutation of base-pairs within a host spacer for two reasons. This is for two reasons. First, selection acts against mutations of host spacers because they weaken spacer/proto-spacer binding and thus antiviral immunity (and by the above infinite alleles argument, hosts are very unlikely to randomly make the same mutations as the viral strains). And even if host spacer mutations were selectively neutral in not reducing CRISPR-spacer binding (e.g., because the Gibbs Free Energy increase is insignificant gifrom single base-pair mutations), it would take these neutral mutations an average of N generations to drift to fixation, where N is the large effective population size.

The second and more significant reason for ignoring host spacer mutation is that host genomes (10^6^-10^7^ base-pairs) are orders of magnitude larger than viral genomes (10^3^-10^5^ base-pairs). Yet, larger genomes do not accumulate mutations any quicker than shorter genomes, as Drake et al., have noted [2]. The *per-genome* mutation rate of a DNA-based microbe—virus or host—is effectively constant empirically at approximately .003 mutations per replication [2]. Thus, the probability of mutating any spacer within a CRISPR locus is two to three orders of magnitude lower than the probability of mutating any proto-spacer within a virus.

**vi) Host Spacer Deletions Only Occur During Additions**

As noted in the main text, the model assumption that spacer deletion only occurs when a strain adds spacers is consistent with experimental data. The current hypothesized mechanism by which CRISPR loci delete spacers is homologous recombination [10,11], which should result in spacers being added and deleted from a strain concomitantly. To that end, spacer deletions are often observed on strands exhibiting recent spacer additions[4]. Further linking the genomic insertion and deletion processes of microbes, Palmer and Gilmore captured an example in which the CRISPR locus of an Enterococcus line is deleted during the incorporation of an adjacent genomic island [12]. Coupled insertions and deletions may be a key mechanism by which prokaryotes keep their genome lengths below an empirical threshold of approximately 13 MB, despite continually updating their genomes with new horizontally transferred regions [13]. Finally, a coupled addition and deletion process may also explain why deleted spacers are frequently empirically observed despite small deletions not providing hosts much in the way of selection benefits.

**vii) Mutants Initialized at Fraction of Parent Strain Frequencies**

We initialized new host and viral mutants at a moderate fraction (parameterized at 10%) of their parent strains’ frequency to prevent fit variants from immediately being lost due to model clearance of low-frequency strains. But what allows us to assume that beneficial mutants initially avoid genetic drift and converge to parent-based frequencies in their first generation? As we noted in ignoring sampling noise in the interaction step of the model, the justification is twofold: a) the experimentally demonstrated strength of selection for immune hosts and infective viruses in CRISPR-virus interactions and b) the extremely large microbial and phage effective population sizes (i.e., depressed genetic drift).

In a large population, the probability of a new mutant establishing (i.e., avoiding extinction when first created) increases as its selection advantage increases. This was shown by Kimura, who approximated the probability, P, of a new mutant avoiding extinction as **P = (1-e^-2s^)/(1-e^-2sN^)**, where N is the population size and s the selection coefficient of the mutant[14]. With N large as in our microbe-phage system and s>>1/N, the probability of a new mutant establishing is **P = 1-e^-2s^** (note that this is sometimes estimated in the literature as P≈2s, an assumption that holds under weak to moderate selection [5]). Because CRISPR-virus systems have been measured to be under extremely strong selection [3,4], the above equation predicts that highly beneficial host and viral mutants are likely to be established in large populations due to selection dwarfing drift. For simplicity, we allow all mutants to establish. Importantly mutant establishment only allows new mutants to compete in the clonal interference step of the model. If the established mutants do not offer increased fitness (for example, viruses which mutate proto-spacers not incorporated by any hosts have no selection advantage), they will be quickly be outcompeted by fitter clones and cleared.

Given the decision to establish all new mutants, the relevant question is then what the establishment probability should be. In the model, host and viral frequencies are determined directly from the relative fitnesses of the strains. Moreover, new mutants inherit most of their fitness form their parents, having only changed a single spacer or proto-spacer. Thus, we initialized mutants at a fraction of their parents’ frequency (generally 10%, but varied). The reason mutants were not allowed to rise to the full value of their parents’ fitness during long model iterations is that mutants initially start extremely small. Indeed in a fuller model that includes drift and the initialization of new mutants at frequency 1/N, new mutants would rise to the frequency (1/N)*(1+s) after a single iteration. With s largely inherited, this effectively puts mutants at a fraction of their parents’ frequency.

**viii) Frequency Thresholds Below Which Strains Are Cleared, Excepting New Mutants in ‘Grace-Periods’.**

To effect mutation-selection balance in the model and to prevent a limitless buildup of low-frequency, unfit strains, the model clears ‘old’ strains whose frequencies have dipped below a threshold due to lack of fitness. At the same time, to prevent fit new mutants from being cleared before selection has the chance to increase their frequencies above the clearance threshold, each new mutant is given a clearance-free ‘emergence period,’ during which the mutant is not subject to model clearance even when low in frequency. Importantly, removing the emergence period does not substantively alter long-run model dynamics (Supplementary Fig. 9).

The implicit assumption in this step is that while virus and host population sizes remain large throughout a simulation, the particular virus and host strains making up these large populations turnover rapidly. This assumption is directly supported by the conclusions of the above metagenomic tracking experiment in four aquatic environments. That study found high-level mutational turnover at the ‘strain’ (genotypic) level of virus and host coupled with the above-mentioned stability at the ‘species’ (i.e., total abundance) levels[1].

**Alternative Model Formulations**

While we employed a frequency-based population-genetic model with discrete, non-overlapping generations, one envisions alternative modeling paradigms that combine ecologically dependent virus-host population dynamics with random host and viral mutation. Such ‘eco-evolutionary’ models have been pursued in well-studied systems such as HIV-T cell dynamics in human hosts [15]; here we describe why metagenomic, natural systems data are not yet ripe for such fine-scale modeling.

In order to completely include population dynamics in a coevolutionary model, one needs to understand both the ecological dynamics of the environment being simulated (e.g., how frequently nutrients are being pumped into and out of a system) and the kinetics through which virus and host populations increase, decrease and interact (e.g., how well such nutrients are utilized by the microbes). And even if one had measurements of the extrinsic, environmental variables, the results would almost certainly be difficult to generalize to new, unstudied environments. More critically, one presently cannot measure the relevant rates of virus-host growth and interaction, because naturally occurring microbes are generally unculturable in the laboratory. In fact, a major utility of metagenomics is that it allows us to get snapshots of otherwise unobservable microbes [16]. But these snapshots only offer relative, frequency-based information about virus-host dynamics, given that metagenomics is a sampling-based technique. As in our model, one can metagenomically infer a viral bloom but never metagenomically quantify how many viruses actually bloomed. That is why we employed a frequency-based framework.

**References**

1. Rodriguez-Brito B, Li L, Wegley L, Furlan M, Angly F, et al. Viral and microbial community dynamics in four aquatic environments. ISME J 4: 739-751.

2. Drake JW, Charlesworth B, Charlesworth D, Crow JF (1998) Rates of spontaneous mutation. Genetics 148: 1667-1686.

3. Barrangou R, Fremaux C, Deveau H, Richards M, Boyaval P, et al. (2007) CRISPR provides acquired resistance against viruses in prokaryotes. Science 315: 1709-1712.

4. Deveau H, Barrangou R, Garneau JE, Labonte J, Fremaux C, et al. (2008) Phage response to CRISPR-encoded resistance in Streptococcus thermophilus. J Bacteriol 190: 1390-1400.

5. Barrett RD, M'Gonigle LK, Otto SP (2006) The distribution of beneficial mutant effects under strong selection. Genetics 174: 2071-2079.

6. Wilson JB (1990) Mechanisms Of Species Coexistence: Twelve Explanations For Hutchinson's 'Paradox Of The Plankton': Evidence From New Zealand Plant Communities. New Zealand Journal of Ecology 13: 17-42.

7. Rainey PB, Travisano M (1998) Adaptive radiation in a heterogeneous environment. Nature 394: 69-72.

8. Marraffini LA, Sontheimer EJ (2010) Self versus non-self discrimination during CRISPR RNA-directed immunity. Nature 463: 568-571.

9. Kimura M, Crow JF (1964) The Number of Alleles That Can Be Maintained in a Finite Population. Genetics 49: 725-738.

10. Horvath P, Barrangou R (2010) CRISPR/Cas, the immune system of bacteria and archaea. Science 327: 167-170.

11. Marraffini LA, Sontheimer EJ (2010) CRISPR interference: RNA-directed adaptive immunity in bacteria and archaea. Nat Rev Genet 11: 181-190.

12. Palmer KL, Gilmore MS (2010) Multidrug-Resistant Enterococci Lack CRISPR-cas. MBio 1.

13. Kuo CH, Ochman H (2009) Deletional bias across the three domains of life. Genome Biol Evol 1: 145-152.

14. Kimura M (1964) Diffusion models in population genetics J Appl Probability 1: 177–232

15. Althaus CL, De Boer RJ (2008) Dynamics of immune escape during HIV/SIV infection. PLoS Comput Biol 4: e1000103.

16. Schleper C (2008) Microbial ecology: Metabolism of the deep. Nature 456: 712-714.
