# Supplementary material for: Persisting Viral Sequences Shape Microbial CRISPR-based Immunity
Source: PLoS Comput Biol. 2012 Apr 19;8(4):e1002475. doi: 10.1371/journal.pcbi.1002475 (PMC3330103; doi:10.1371/journal.pcbi.1002475)
Supplement: Text S3 — Algorithm of optimal clustering routine. To track host and viral genetic diversity across model iterations, we developed a silhouette-based clustering routine. In a given model iteration, the clustering routine distributes host and viral populations into a chosen ‘optimal’ number of sub-populations. Text S3 describes how this optimal number is chosen and how strains are then divided into their respective sub-populations. (DOC) [file pcbi.1002475.s012.doc]

**Text S3 Weinberger et al.,**

Silhouette-based Clustering Algorithm

To quantify how host diversity changes across time, computationally generated loci were clustered into an optimal number of sub-populations at frequent iterations. The partitioned sub-populations and their progeny were then tracked across time. Results show how diversity is abruptly lost during selective sweeps (see the 3800th iteration in Fig. 2B), after which diversity is regained coincident with new-end diversifications within the sweeping sub-population (see the final thousand iterations in Fig. 2B and fig. S3). The regained host diversity is captured because the clustering algorithm compares host strains at all locus positions in determining an optimal number of sub-populations for an iteration. This is in contrast to the spacer dynamics plots (*e.g.*, Fig. 2C and Fig. S2), which show diversity at a single locus position across time. That locus position will eventually become clonal in the absence of deletions.

*Determining an Optimal Number of Clusters.* The clustering algorithm begins by determining the number of clusters in the first snapshot (*T*=100). To do so, the model counts the number of distinct spacers at each locus position (column) of the aligned host strains in the snapshot. Because the number of viral proto-spacers is large—there are approximately 900 proto-spacers at steady state when the viral genomes are set to have 50 proto-spacers—and hosts sample relatively infrequently (1-10 times per iteration) from this list, the algorithm assumes that two different host strains will not incorporate the same spacer. Thus, when two hosts share the same spacer at a given locus position, the algorithm assigns them to the same lineage and assumes all previous locus positions have the same spacers as well.

For each locus column in the snapshot, an independent clustering diagram is generated by simply dividing the hosts into sub-populations based on their spacer in that column. In other words, if column 2 only contains the spacers 3 and 4, all hosts with the spacer 3 in column 2 are placed into one cluster and all hosts with the spacer 4 in column 2 are placed into the other cluster. Using the Hamming distance, which calculates the proportion of shared elements (spacers) in two aligned strains, we were able to determine an average silhouette width [1] for each clustering diagram (*i.e.*, for each column). The silhouette is a cluster validation technique, which gives a value of how well-clustered a population is. By maximizing this metric, the column that optimally partitions the first snapshot’s host strains by lineage is chosen. Subsequent snapshots are clustered in the same way, with the exception that only newer columns (*i.e.*, to the left of previous clustering columns) are compared for a maximal silhouette width. With the column upon which clustering is calculated always to the left of clustering columns from previous iterations, we are able to determine parent clusters for each cluster by checking their spacer in the previous iteration’s clustering column.

*Representing Clusters Across Time.* After making all clusters at each snapshot, the algorithm colors from the final snapshot backwards. All distinct clusters with no progeny are assigned distinct random colors (to maximize the contrast between distinct clusters). Parent clusters are then given the average color of their progeny clusters. In each iteration, the clusters are then displayed. Each cluster’s height reflects the summed frequency of all strains within it and the clutser’s width reflects the total number of strains in that cluster. The combined height of all clusters in an iteration represents the fraction of virus-host interactions that is immune.

**References**

1. Rousseeuw PJ (1987) Silhouettes: A graphical aid to the interpretation and validation of cluster analysis. J Comput Appl Math 20: 53-65.
